# Imaging mass cytometry analysis of Becker muscular dystrophy muscle samples reveals different stages of muscle degeneration

**DOI:** 10.1038/s41598-024-51906-x

**Published:** 2024-02-09

**Authors:** Patricia Piñol-Jurado, José Verdú-Díaz, Esther Fernández-Simón, Cristina Domínguez-González, Aurelio Hernández-Lain, Conor Lawless, Amy Vincent, Alejandro González-Chamorro, Elisa Villalobos, Alexandra Monceau, Zoe Laidler, Priyanka Mehra, James Clark, Andrew Filby, David McDonald, Paul Rushton, Andrew Bowey, Jorge Alonso Pérez, Giorgio Tasca, Chiara Marini-Bettolo, Michela Guglieri, Volker Straub, Xavier Suárez-Calvet, Jordi Díaz-Manera

**Affiliations:** 1 John Walton Muscular Dystrophy Research Centre, Newcastle University Translational and Clinical Research Institute, Center for Life, Central Parkway, Newcastle Upon Tyne, NE13BZ UK; 2 Neuromuscular Disorders Unit, Neurology Department, imas12 Research Institute, Hospital Universitario, 12 de Octubre, Madrid, Spain; 3 Centro de Investigación Biomédica en Red en Enfermedades Raras (CIBERER), Barcelona, Spain; 4 Neuropathology Unit, imas12 Research Institute, Hospital Universitario, 12 de Octubre, Madrid, Spain; 5 Translational and Clinical Research Institute, Newcastle University, Newcastle, UK; 6 Faculty of Medical Sciences, Welcome Centre for Mitochondrial Research, Translational and Clinical Research Institute, Newcastle University, Newcastle Upon Tyne, UK; 7 Newcastle University Biosciences Institute and Innovation Methodology and Application Research Theme, Newcastle University, Newcastle Upon Tyne, UK; 8 Department of Orthopaedic Spine Surgery, Great North Children’s Hospital, Royal Victoria Infirmary, Newcastle Upon Tyne, UK; 9 Neuromuscular Disease Unit, Neurology Department, Hospital Universitario Nuestra Señora de Candelaria, Fundación Canaria Instituto de Investigación Sanitaria de Canarias (FIISC), Tenerife, Spain; 10 Neuromuscular Diseases Unit, Department of Neurology, Hospital de la Santa Creu i Sant Pau, Institut d’Investigació Biomèdica Sant Pau (IBB SANT PAU), Barcelona, Spain

**Keywords:** Bioinformatics, Biological models, Immunological techniques, Mass spectrometry, Microscopy, Software

## Abstract

Becker muscular dystrophy (BMD) is characterised by fiber loss and expansion of fibrotic and adipose tissue. Several cells interact locally in what is known as the degenerative niche. We analysed muscle biopsies of controls and BMD patients at early, moderate and advanced stages of progression using Hyperion imaging mass cytometry (IMC) by labelling single sections with 17 markers identifying different components of the muscle. We developed a software for analysing IMC images and studied changes in the muscle composition and spatial correlations between markers across disease progression. We found a strong correlation between collagen-I and the area of stroma, collagen-VI, adipose tissue, and M2-macrophages number. There was a negative correlation between the area of collagen-I and the number of satellite cells (SCs), fibres and blood vessels. The comparison between fibrotic and non-fibrotic areas allowed to study the disease process in detail. We found structural differences among non-fibrotic areas from control and patients, being these latter characterized by increase in CTGF and in M2-macrophages and decrease in fibers and blood vessels. IMC enables to study of changes in tissue structure along disease progression, spatio-temporal correlations and opening the door to better understand new potential pathogenic pathways in human samples.

## Introduction

Becker muscular dystrophy (BMD) is a X-linked disorder produced by mutations in the dystrophin gene causing progressive muscle weakness leading to severe disability and cardio-respiratory complications^1^. Dystrophin is a subsarcolemmal protein linking muscle fiber to the extracellular matrix (ECM) through an interaction with the sarcoglycan-dystroglycan complex^2^. The main role of dystrophin is to stabilize fiber membrane during muscle contraction preventing contraction-induced damage. Muscle fibers from patients with BMD, that expressed a low amount of dystrophin, are injured during muscle contraction, leading to continuous cycles of fiber regeneration and degeneration^3^. In the long term, muscle fibers are lost and progressively substituted by fibrotic and fat tissue. The mechanisms involved in the process of muscle degeneration in humans are not completely understood. It has been established that this process requires the participation of many different cell types including damaged muscle fibers, satellite cells (SCs), fibroadipogenic progenitor cells (FAPs) and inflammatory cells, such as macrophages that interact locally in what is known as the degenerative niche^4^. SCs are the main stem cell of the muscle responsible of regenerating damaged muscle fibers^5^. After an acute injury, they activate, proliferate, and differentiate into myoblasts fusing with the injured fibers^6^. However, in muscular dystrophies such as BMD these cells fails to efficiently regenerate skeletal muscle by a mechanism that is not completely known, although it is thought to be related with an the existence of an hostile microenvironment displaying chronic inflammation, fat and fibrosis impairing SCs’ differentiation capacity^7^. FAPs have been described to play a leading role in the process of both regeneration and degeneration^8^. After an acute damage, FAPs are activated and proliferate releasing pro-regenerative factors that influence SCs function and ECM that serves as scaffold for the new regenerated fibres^9,10^. Once the muscle fibers are completely recovered, the number of FAPs return to basal through a Tumour Necrosis Factor alpha (TNF-α) mediated apoptosis^11^. In muscular dystrophies FAPs are continuously activated releasing collagens and other component of the ECM leading to muscle fibrosis. Depending on the stimulus received, FAPs can also differentiate into adipocytes which are responsible of the accumulation of fat observed in muscle biopsies of patients^12^. Macrophages are the most frequent inflammatory cells infiltrating the muscle of muscular dystrophy patients, and among them M2 macrophages are thought to release profibrotic cytokines such as Transforming Growth Factor-β (TGF-β) that influence FAPs proliferation and differentiation into fibrotic cells^13^.

Although the process of muscle degeneration has been widely studied in murine models of muscular dystrophies, it is still not known to what extent the process is exactly replicated in humans. In recent years new technologies such a single cell/nuclei RNA sequencing and spatial biology including spatial transcriptomics and imaging mass cytometry (IMC) are allowing the analysis of human samples more efficiently generating a considerable amount of new information about the cellular and molecular mechanisms of different diseases^14,15^. This is especially relevant in the case of muscular dystrophies, as these are rare diseases and the availability of muscle biopsies for research is low, which has decreased even more since the popularization of Next Generation Sequencing for diagnosis. Traditional immunohistochemical and histological methods allow the study of protein distribution and expression at the single-cell and sub-cellular level making them suitable for observing any mosaic pattern of affected and unaffected tissue^16^. However, multiple serial sections are often required to observe large numbers of markers, which has some limitations as it requires large amount of material and makes difficult tracing muscle fibers and cells across serial sections to study potential interactions. IMC is an imaging technique based on Cytometry by time of flight (CyTOF) technology that allows studying up to 40 markers in a single section of tissue^17^.

The main aim of this paper is to analyse the different cell types, ECM proteins, growth factors and markers of muscle regeneration present in muscle biopsies of BMD patients at different stages of disease severity by applying IMC to better understand the process of muscle degeneration in humans.

## Methods

### Patients

Muscle biopsies of BMD patients were obtained for diagnosis and stored at the Biobank of Neuromuscular Diseases of the John Walton Muscular Dystrophy Research Center. Patient’s muscle biopsies were extensively studied using immunohistochemistry and western-blot for diagnosis purposes demonstrating in all cases a reduction of dystrophin expression. The diagnosis of BMD was confirmed genetically. Additional samples for validating the antibodies were obtained from a collaboration with Hospital 12 de Octubre in Madrid (Spain). Muscle biopsies from controls were obtained by the orthopaedic surgeons working at the Newcastle Upon Tyne Foundation Trust from patients undergoing a surgical procedure. Conventional staining was performed to confirm that these latter muscle biopsies were normal. Informed consent was obtained from all subjects to participate in the study. The study was approved by the Ethical Committee of the University of Newcastle and the National Health Service-Health Research Authority, Research Ethics Committee (REC) (reference ID: 19/NE/0028) and it was performed following the ethical standards of Declaration of Helsinki.

### Antibodies and panel design

A panel of 17 antibodies was used as makers for different cells and cellular processes as listed in Table 1. All antibodies used in this paper were obtained in a carrier (BSA)-free buffer. Each antibody was tested first using immunofluorescence (IF) in muscle samples from either controls or patients with muscular dystrophy to determine the relative expression levels based on comparative fluoresce intensity. Based on the data collected we ranked the antibodies and paired them with an appropriate metal for conjugation, ensuring that the targets with a higher expression level based on fluorescent intensity were conjugated to a weaker metal and, those with a lower expression levels based on fluorescent intensity were paired with a stronger antibody metal. In the majority of cases, antibodies were purchased directly conjugated from the supplier (Standard Biotools, San Francisco, CA, USA) as per Table 1. For those antibodies that were not already conjugated to a metalwe used the MaxPar antibody conjugation kit (Standard Biotools) following the manufacturers’ instructions without deviation. Once conjugated, antibodies were suspended in an antibody stabilisation solution [Antibody stabiliser (PBS), Candor Bioscience, Wangen im Allgäu, Germany]. Final concentration of the antibodies in the mixed solution was recalculated using a Nanodrop (Thermo Fisher Scientific, Waltham, MA, USA) and kept at 4 °C util for further use. To test for the successful conjugation, metal conjugated antibodies were incubated with antibody capture beads (AbC-Total compensation beads, Thermo Fisher Scientific) and the presence of the appropriate metal isotope signal was confirmed by Mass Cytometry in suspension mode (Helios system). Data was exported in the form of FCS 3.0 files (Flow Cytometry standard files) and analysed for metal expression levels using FCS express software (Version 7, Dotmatics, Boston, MA, USA)^18^.

**Table 1:** Antibodies used in this project.

Target	Clone	Metal	Work dilution after conjugation	Cell target
TE-7	TE-7	115Ln	1/80	Fibroblast
Alpha-smooth muscle actin*	14A	141Pr	1/25	Smooth muscle cell
Collagen VI	EPR17072	143Nd	1/30	Extracellular matrix
Perilipin	Polyclonal	145Nd	1/60	Adipose tissue
Laminin	4H8-2	149Sm	1/5	Muscle fiber
CD31*	PECAM1	151Eu	1/50	Endothelial cell
Collagen III	EPR17673	152Sm	1/15	Extracellular matrix
TGF beta-1	TB21	154Sm	1/200	Profibrotic cytokine
CD68*	KP1	159 Tb	1/25	Macrophages
PDGFR alpha	Polyclonal	162Dy	1/15	FAPs
CTGF	Polyclonal	166Er	1/10	Profibrotic cytokine
PDGF-AA	Polyclonal	167Er	1/10	Profibrotic cytokine
CD206*	15–2	168Er	1/50	M2 macrophages
Collagen I*	Polyclonal	169Tm	1/50	Extracellular matrix
MYH3	Polyclonal	174Yb	1/100	Regenerative fibers
CD56*	NCAM16.2	176Yb	1/50	Satellite cells / Regenerative fibers
Nucleic acid		191Ir/193Ir		Cell nuclei

*Commercially conjugated antibodies. *FAPs* fibroadipogenic progenitor cells.

### Preparation of samples for IMC

Rapid freezing of skeletal muscle using isopentane cooled with liquid nitrogen was performed to preserve optimal skeletal muscle morphology. Then, serial 7 µm sections were cut with a Leica cryostat (Leica Microsystems, Wetzlar, Germany). Tissue fixation was performed by submerging the slides in acetone for 5 min and washed three times in TBS at room temperature. The UltraCruz Blocking Reagent (Santa Cruz Biotechnology, Dallas, TX, USA), a commercial blocking buffer, the was then applied to the samples for 1 h at room temperature. Tissue sections were then incubated overnight with the solution containing the primary antibodies bound to metals diluted at four degrees. The day after, slides were washed three times in TBS at room temperature. Cell nuclei were stained with Cell-ID™ Intercalator-Ir (Standard Biotools, CAT#201192A) at a 1 in 400 dilution, for 30 min at room temperature. The slides were then washed for 5 min in Milli-Q® water and air dried prior to ablation.

### Imaging mass cytometry

Prior to each sample acquisition, the Hyperion Tissue Imager was calibrated and rigorously quality controlled to achieve reproducible sensitivity based on the detection of 175Lutetium. Briefly, a stable plasma was allowed to develop prior to ablation of a single multi-element-coated “tuning slide” (Standard Biotools). During this ablation, performance was standardised to an acceptable range by optimising system parameters using the manufacture’s “auto tune” application or by manual optimisation of XY settings whilst monitoring 175Lutetium dual counts. Slides were analyzed with the *Hyperion™ Imaging System* (Fluidigm, San Francisco, CA, USA) at the Flow Cytometry Core Facility in Newcastle University. Slides were photographed at low resolution then loaded with image into the IMC system and imaged by an epifluorescent lightsource to generate panoramas. Regions of interest (ROIs) were set on the panoramas in order select the areas of tissue to ablate for downstream analyses. All ablations were performed according to a pre-generated mass template with a laser shot frequency of 200 Hz. MCD image files were exported as multi-page OME-TIFFs (16-bit) using MCD viewer software (Fluidigim, v1.0.560.6 August 12, 2021)^19,20^.

### Analysis of IMC pseudoimages

Pseudoimages generated by IMC have a resolution of 1 pixel per 1 μm^2^, which is determined by the size of the laser spot. These IMC digital pseudoimages were analysed using HIPO software, an interactive visualization tool in python language created by our group for this specific project^21^. HIPO enables the quantification of the signal obtained from different channels using image stacks, to quantify the percentage of positive area occupied by the signal. The ROI (region of interest) for each sample was drawn using QuPath (version 0.4.3) an image analysis software whose files can be exported in .json format and imported into HIPO^22^. We used napari software (version 0.4.18) to apply thresholds to each channel and sample^23^. For each ROI we calculated the total size of the ROI, the positive fraction defined as percentage of positive pixels for each channel in the ROI, mean intensity of all the pixels, and mean intensity of the positive signal. HIPO was also used to quantify the number of cells or blood vessels using specific cell markers in a semiautomatic way. Muscle fiber segmentation was performed in collaboration with an external company (GAMAED, Newcastle Upon Tyne, UK) using a deep learning approach on the laminin channel. The number, size and marker value of the fibers was extracted using the fiber segmentation and HIPO. All the analysis were performed on a large ROI covering almost the whole sample except the edges, as well as smaller areas of fibrotic foci and areas without apparent fibrosis in the muscles.

### Quantification and statistical analysis

Statistics and bar graphs were prepared using GraphPad Prism v.9 software. Arithmetic means for each experimental cohort were plotted and error bars represented the standard deviation (SD). We used Shapiro-Willis test to confirm that variables for the analysis were distributed following a normal distribution. Statistical significance was assessed using unpaired t tests and significance was reached when the *p* value was ≤ 0.05. The number of replicates (n) for each analysis is reported in the figure captions and refers to the number of samples or the number of areas of interest. Histograms displaying fitted lines for myofiber spreads and cumulative frequency distribution functions were prepared using GraphPad, with significance determined by a Komolgorov-Smirnov (KS) test. Spearman’s |r| was used to study the association of initial levels of each marker with other markers throughout the course of the disease degeneration. As multiple correlations were performed, posthoc Bonferroni correction was applied.

## Results

### Patients

We included in the study muscle biopsies from eight BMD patients and two controls. Demographic, genetic, and clinical data are summarized in Table 2. Haematoxylin–eosin staining of all samples is shown in supplemental Fig. 1.

**Table 2:** Demographic, genetic and clinical data of the patients whose muscle biopsies were included in the study.

Number	Age of onset (Y)	Muscle biopsied	Age at biopsy (Y)	Gender	Mutation	Degree of involvement
Control 1	–	Quadriceps	36	Male		
Control 2	–	Quadriceps	6	Male		
BMD 1	16	Deltoid	24	Male	Deletion exon 45 to 48	Mild
BMD 2	Adolescence	Quadriceps	60	Male	Deletion exon 48 and 49	Mild
BMD 3	12	Quadriceps	28	Male	Deletion exon 48 and 49	Moderate
BMD 4	5	Quadriceps	9	Male	c.10454delT exon 74	Moderate
BMD 5	20	Tibialis Anterior	23	Male	Duplication exons 13 to 29	Advanced—fibrosis
BMD 6	4	Quadriceps	12	Male	Deletion exon 5 to 26	Advanced – fibrosis
BMD 7	50	Quadriceps	53	Male	Deletion exon 14	Advanced – fat replacement
BMD 8	24	Quadriceps	57	Male	Deletion exon 45 to 48	Advanced – fat replacement

### General description of the samples

**Figure 1:**
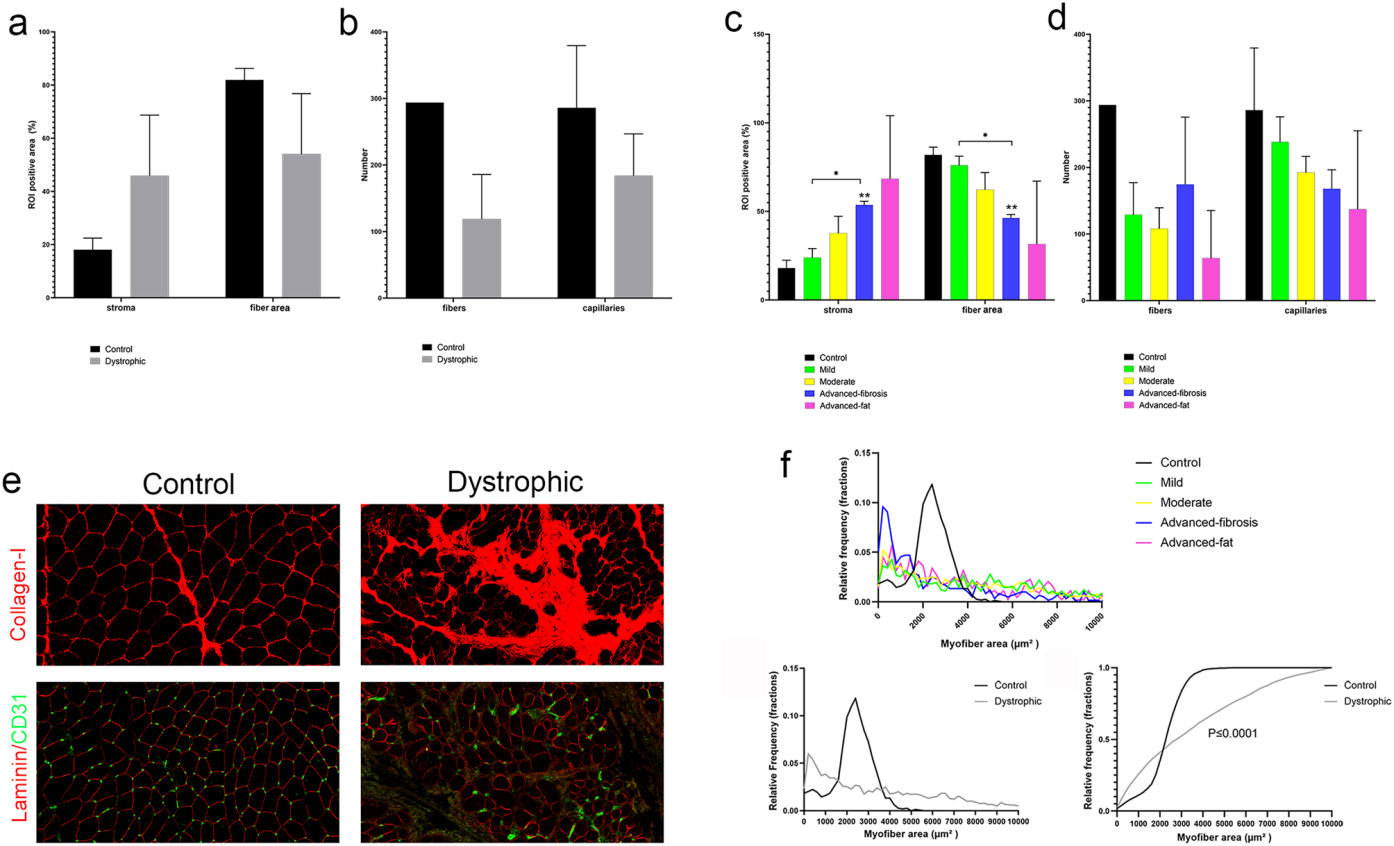
General description of images obtained by IMC and differences between healthy control and dystrophic samples. (**a**) Mean of the positive area (%) occupied by the stroma and fibers in healthy (n = 2, *black bars*) and dystrophic patients (n = 8, *grey bars*). (**b**) Mean of fiber and capillary number in healthy (n = 2, *black bars*) and dystrophic patients (n = 8, *grey bars*). (**c**) Mean of the positive area (%) occupied by the stroma and fibers according to the level of severity of the disease: healthy controls (n = 2, *black bars*), mild (n = 2, *green bars*), moderate (n = 2, *yellow bars*), advanced-fibrosis (n = 2, *blue bars*) and advanced-fat conditions (n = 2, *pink bars*). (**d**) Mean of fiber and capillary number according to the level of severity of the disease: healthy control (n = 2, *black bars*), mild (n = 2, *green bars*), moderate (n = 2, *yellow bars*), advanced-fibrosis (n = 2, *blue bars*) and advanced-fat conditions (n = 2, *pink bars*). (**e**) IMC images of areas occupied by collagen I (*red*) on the top in control and dystrophic patients. Long arrow point to collagen-I located between fibers in normal muscle, while there is a increased collagen deposition in dystrophic patients (long arrow). The bottom images show blood vessels (CD31 + cells, *green,* arrrowheads) between fibers that can be identified thanks to the statining with laminin (*red*) in control and dystrophic samples. Control and dystrophic sample correspond to samples C-1 and BMD-6 (advanced-fibrosis), respectively. (**f**) Top: Relative frequency histogram showing the quantification of myofiber cross-sectional areas (CSAs) in the different study groups: healthy control (n = 2, *black bars*), mild (n = 2, *green bars*), moderate (n = 2, *yellow bars*), advanced-fibrosis (n = 2, *blue bars*) and advanced-fat conditions (n = 2, *pink bars*). Bottom left: Relative frequency graph showing the quantification of CSAs from each group: healthy group (n = 2, *black line*) and dystrophic patients group (n = 8, *grey line*). Bottom right: An empirical cumulative distribution function graph of all myofiber CSAs from the noted groups, with significance determined by a KS test (n = 1572 [healthy control group] and 3978 [dystrophic patients]). Error bars represent ± standard deviation (SD). *p ≤ 0.05, **p ≤ 0.01, ***p ≤ 0.001.

We analysed the expression of our markers of interest in the whole muscle section using Hyperion (Table 1). As shown in Fig. 1, the amount of stroma increased with disease progression while the space occupied by muscle fibers decreased. However, there was not a correlation between the area occupied by stroma and the number of fibers normalized by the area of the muscle. The total number of capillaries was reduced along disease progression and correlated negatively with the area of the stroma (R: − 0.72, *p* < 0.01, supplemental Fig. 2) although it was not influenced by the number of remaining fibers (Fig. 1b–d). We detected an increased variability in the cross-sectional area (CSA) of muscle fibers in dystrophic patients compared to controls (3048 ± 3464 µm^2^ in BMD patients vs. 2295 ± 900 µm^2^ in controls) (Fig. 1f–h).

### Components of the stroma in dystrophic versus control muscle samples

**Figure 2:**
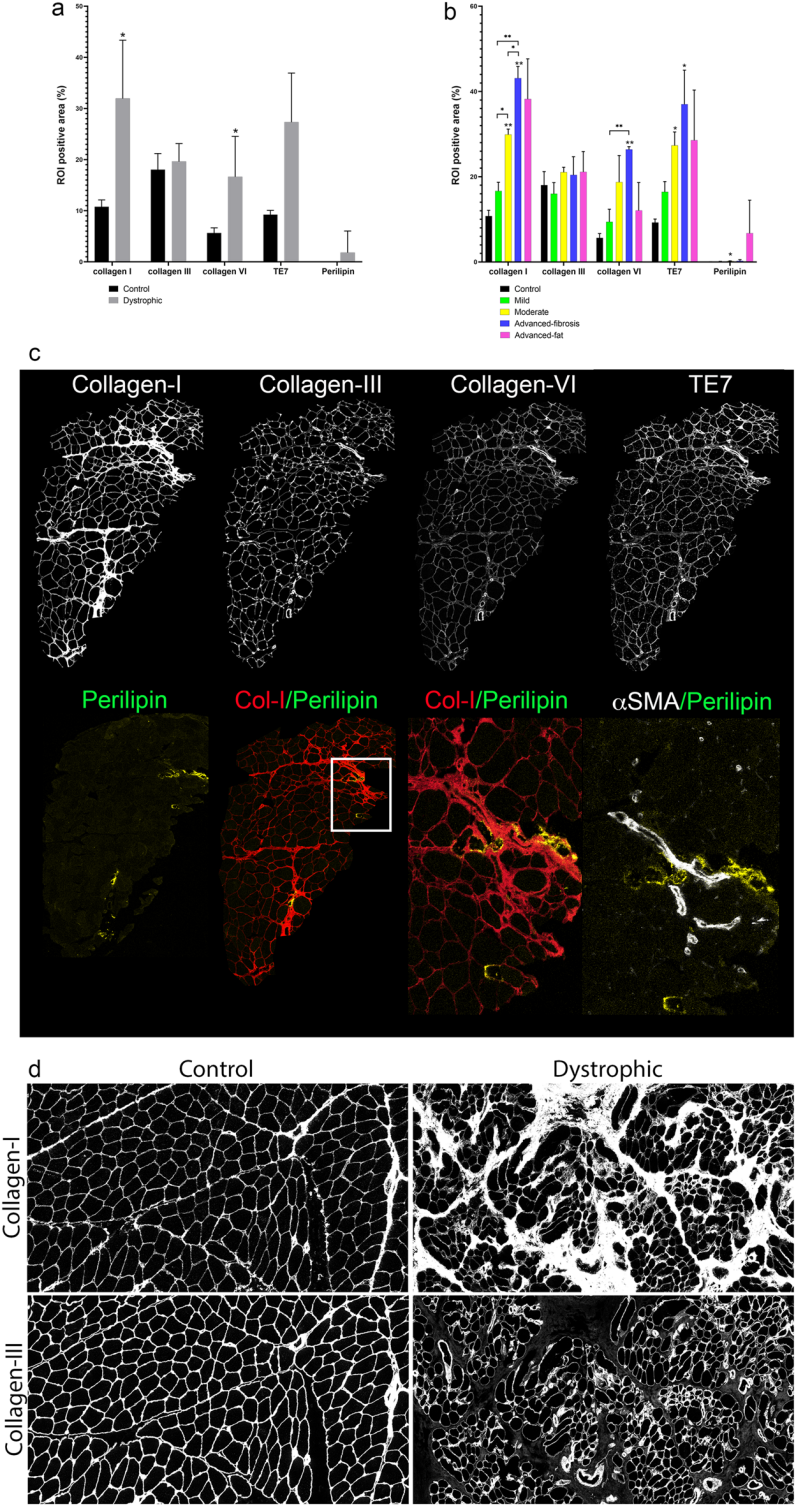
ECM proteins in dystrophic and control muscle samples. (**a**) Mean of the ROI positive area (%) occupied by collagen I, collagen III, collagen VI, TE7 and perilipin in healthy controls (n = 2, *black bars*) and dystrophic muscles (n = 8, grey bars). (**b**) Mean of the ROI positive area (%) occupied by collagen I, collagen III, collagen VI, TE7 and perilipin in healthy controls (n = 2, *black bars*), mild (n = 2, *green bars*), moderate (n = 2, *yellow bars*), advanced-fibrosis (n = 2, *blue bars*) and advanced-fat conditions (n = 2, *pink bars*). (**c**) IMC images showing collagen I, collagen III, collagen VI, TE-7 and perilipin staining in the muscle sample from BMD-2 (mild condition). Yellow arrows points to perilipin accumulation in close localization to collagen-I deposition (red arrow) and to vessels expressing αSMA (white arrow). (**d**) IMC images of a control (left) and BMD-6 sample (advanced fibrosis-right) showing examples of collagen I and collagen III expression. Yellow arrows show normal accumulation of collagen I and III in the stroma between fibers in controls. Blue arrows point to the abnormal extensive accumulation of collagen-I occupying the stroma of the dystrophic patient, while collagen-III expression remains at a similar level than in control surrounding muscle fibers. Error bars represent ± SD. *p ≤ 0.05, **p ≤ 0.01, ***p ≤ 0.001.

Collagen-III was the main component of the stroma in controls surrounding muscle fibers and vessels (Fig. 2a). In patients, we observed a progressive increase in the amount of collagen-I and VI (Fig. 2b) (R: 0.77, *p* = 0.01, supplemental Fig. 2), but not in collagen III that remained stable (Fig. 2a,b,d). Staining of Human Thymic Fibroblasts Antibody (TE7), a marker classically used to detect fibroblasts^24^, was widespread in the stroma coinciding and correlating with collagen-I and VI deposition (R: 0.95 (*p* < 0.001) and R: 0.78 (*p* = 0.01), respectively). We did not detect perilipin, a marker of fat vacuoles membrane, in control samples, while it was detected in dystrophic samples, especially in advanced stages of the disease (Fig. 2a,b). The area of perilipin expression correlated significantly with the area occupied by stroma (R: 0.78, *p* = 0.01), collagen-I (R: 0.70, *p* = 0.03) and TE7 (R: 0.66, *p* = 0.04). Interestingly, we observed foci of fibrosis in the muscle samples that contained collagen-I and collagen-VI (Fig. 2d). The same was observed with perilipin, which was found embedded in the fibrotic areas closely located to big vessels (Fig. 2c).

### Different cell populations are present in muscle samples

**Figure 3:**
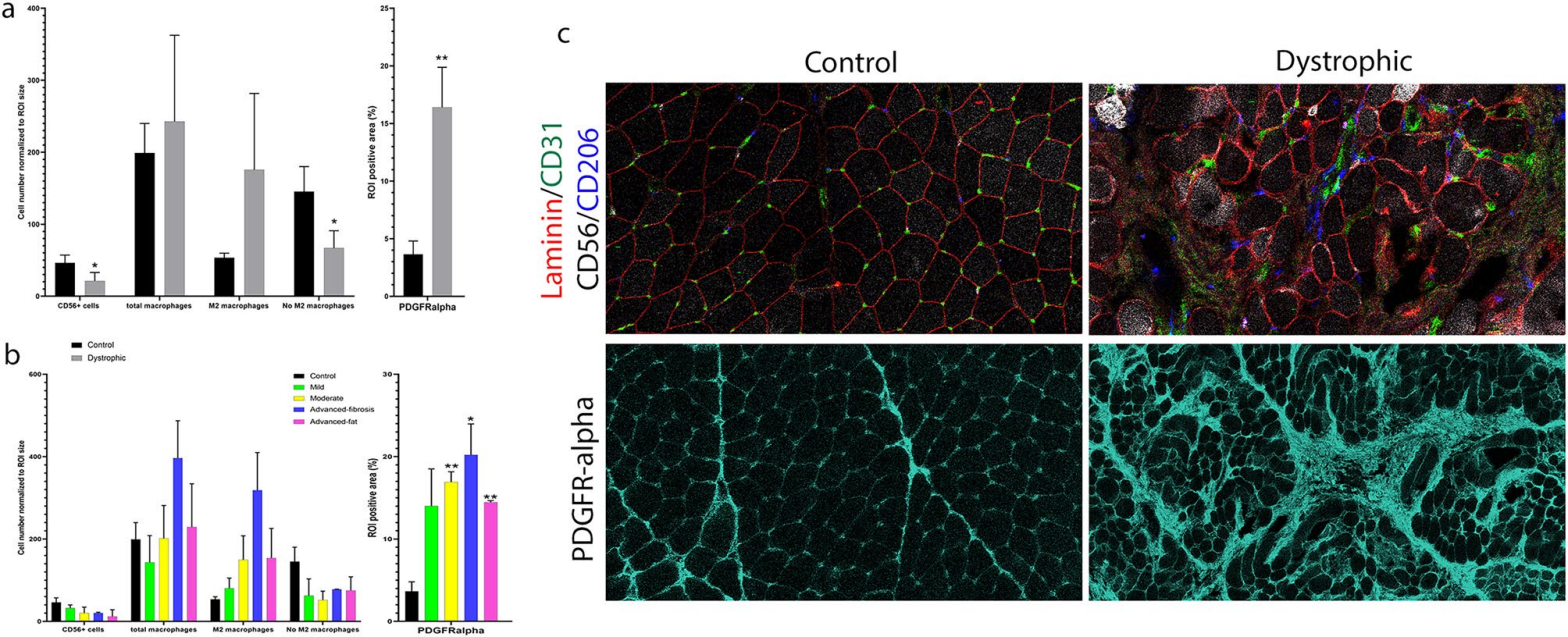
Different cell type found in the muscles of study. (**a**) Mean of CD56 + cell, macrophage (CD68 +), M2 macrophage (CD206 +) and non-M2 macrophage (CD68 + CD206-) number and ROI positive area (%) occupied by PDGFRalpha (FAP marker) in healthy controls (n = 2, *black bars*) and dystrophic muscles (n = 8, grey bars). (**b**) Mean of CD56 + cell, macrophage (CD68 +), M2 macrophage (CD206 +) and non-M2 macrophage (CD68 + CD206-) number and ROI positive area (%) occupied by PDGFRalpha (FAP marker) according to the level of severity of the disease: healthy controls (n = 2, *black bars*), mild (n = 2, *green bars*), moderate (n = 2, *yellow bars*), advanced-fibrosis (n = 2, *blue bars*) and advanced-fat conditions (n = 2, *pink bars*). (**c**) IMC images of C-1 (control) and BMD-6 sample (advanced-fibrosis condition) showing M2 macrophages (CD206 + , *blue*), capillaries (CD31 + , *green*), CD56 + cells (*white*) and laminin (*red*) at the top of the panel and PDGFR + cells (*cyan*) at the bottom of the panel. Blue arrows point to macrophages showing a clear increase in their number in the dystrophic sample. Yellow arrows point to capillaries. As shown in figure, macrophages (blue arrows) accumulate close vessels (yellow arrow) in the dystrophic sample. Green arrow points to PDGFR-alpha staining (FAPs) located in the interfiber space and clearly increased in the dystrophic sample. Error bars represent ± SD. *p ≤ 0.05, **p ≤ 0.01, ***p ≤ 0.001.

As previously mentioned, muscle degeneration is regulated by many different cells, including muscle fibers, SCs, macrophages and, FAPs^4^. We analyzed the presence of these cell populations as well as their variation in number along disease progression (Fig. 3a,b). We observed a progressive decrease in the total number of SCs along with an increase in the stroma area (R: − 0.79, *p* < 0.01). The number of SCs did not correlate with the number of remaining muscle fibers (R: 0.44, *p* = 0.20), but interestingly, it correlated with the number of capillaries (R: 0.77, *p* = 0.01). In this sense, many of the SCs were found close to the vessels especially in the control samples, but that was not the case in dystrophic patients, where we observed an increased number of SCs located far from the capillaries, suggesting that once activated, SCs can migrate to areas of muscle damage (Supplemental Fig. 3). The number of macrophages increased progressively throughout muscle degeneration, and this was mainly relying on an increase of M2 macrophages (positive for CD206) that correlated positively with the area of the stroma (R: 0.78, *p* = 0.01, supplemental Fig. 2) and perilipin area (R: 0.65, *p* = 0.049), and negatively with the number of SCs (R: − 0.64, *p* = 0.05). As observed with SCs, macrophages were often seen close to capillaries in controls, while in patients, there were more often seen away from the vessels infiltrating the stroma (Supplemental Fig. 4). To assess the number of FAPs, we tested several antibodies against Platelet Derived Growth Factor Receptor Alpha (PDGFRα) (Fig. 3c). The signal observed was similar to the one observed with TE7, so a widespread staining of the stroma rather than identifying single cells. We observed a statistical trend in the correlation between PDGFRα area, the stroma (R: 0.62, *p* = 0.06), and the number of M2 macrophages (R: 0.84, *p* < 0.01).

### Markers of muscle regeneration/degeneration and profibrotic cytokines

**Figure 4:**
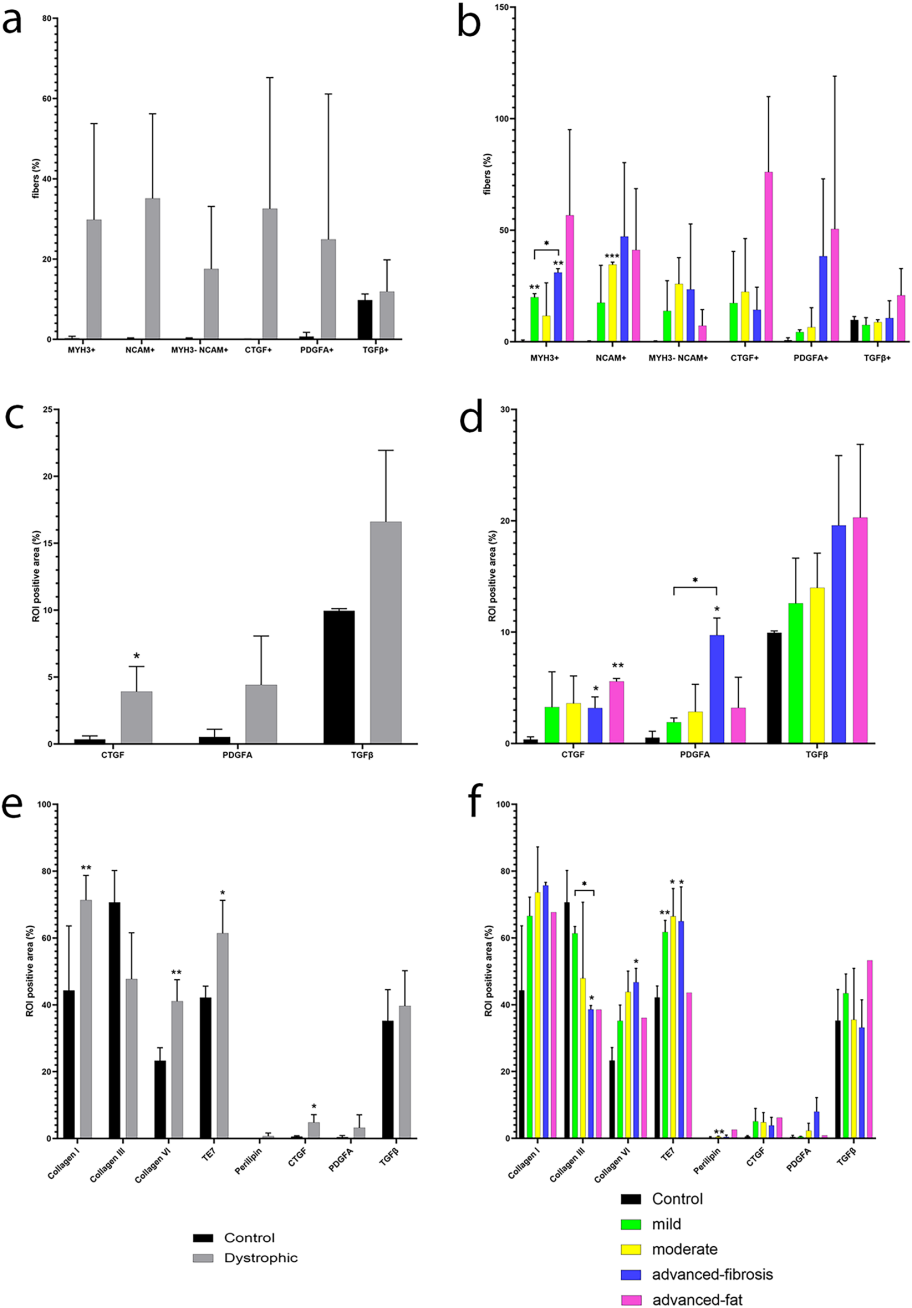
Markers of regeneration/degeneration and growth factors. (**a**) Percentage of MYH3 + , CD56 + , MYH3-CD56 + , CTGF + , PDGFA + , and TGFβ + fibers using a threshold of 10% (fiber is positive for at least 10% of its area), 5% in the case of CTGF, in healthy controls (n = 2, *black bars*) and and dystrophic muscles (n = 8, grey bars). (**b**) Percentage of MYH3 + , CD56 + , MYH3-CD56 + , CTGF + , PDGFA + , and TGFβ + fibers using the same thresholds as before in healthy controls (n = 2, *black bars*), mild (n = 2, *green bars*), moderate (n = 2, *yellow bars*), advanced-fibrosis (n = 2, *blue bars*) and advanced-fat conditions (n = 2, *pink bars*) after fiber segmentation. (**c**) Mean of the positive area (%) occupied by CTGF, PDGFA and TGFβ in healthy controls (n = 2, *black bars*) and dystrophic muscles (n = 8, grey bars). (**d**) Mean of the positive area (%) occupied by CTGF, PDGFA and TGFβ in healthy controls (n = 2, *black bars*), mild (n = 2, *green bars*), moderate (n = 2, *yellow bars*), advanced-fibrosis (n = 2, *blue bars*) and advanced-fat conditions (n = 2, *pink bars*). (**e**) Mean of the ROI positive area (%) occupied by collagen I, collagen III, collagen VI, TE7, perilipin, CTGF, PDGFA and TGFβ in the muscle stroma of healthy controls (n = 2, *black bars*) and dystrophic muscles (n = 8, grey bars) after segmentation. (**f**) Mean of the ROI positive area (%) occupied by collagen I, collagen III, collagen VI, TE7, perilipin, CTGF, PDGFA and TGFβ in the muscle stroma of healthy controls (n = 2, *black bars*), mild (n = 2, *green bars*), moderate (n = 2, *yellow bars*), advanced-fibrosis (n = 2, *blue bars*) and advanced-fat conditions (n = 2, *pink bars*) after segmentation. Error bars represent ± SD. *p ≤ 0.05, **p ≤ 0.01, ***p ≤ 0.001.

To identify regenerating muscle fibers we used an antibody against myosin heavy chain three (MYH3 + , neonatal myosin). We observed an increase in the percentage of fibers that were MYH3 + already at early stages of muscle damage (Fig. 4a,b). The ratio of MYH3 + fibers increased progressively throughout the process of muscle degeneration (Fig. 4b) and correlated with the number of SCs (R: − 0.76, *p* = 0.02, Supplemental Fig. 5), area of the stroma (R: 0.89, *p* = 0.001) and the number of M2 macrophages (R: 0.74, *p* = 0.01). We also assessed the number of fibers expressing CD56/NCAM1 which is expressed by denervated muscle fibers^25^. Most of the MYH3 fibers were co-expressing NCAM, but we observed a considerable number of NCAM + fibers that were not expressing MYH3 as shown in Fig. 4a,b.

We analysed the expression of some growth factors that have been related to the process of fibrosis, such as TGFβ, connective tissue growth factor (CTGF) and Platelet Derived Growth Factor-AA (PDGF-AA) in the muscle sample including the stroma and the muscle fibers. We observed an increase in CTGF and PDGFAA levels in the dystrophic muscles compared to controls but not in TGFβ (Fig. 4c,d). CTGF levels correlated with the number of SCs (R: − 0.70, *p* = 0.03) and perilipin area (R: 0.72, *p* = 0.02, supplemental Fig. 2) but not with the area of the stroma or collagen levels. PDGF-AA levels correlated with the area of the stroma (R: 0.67, *p* = 0.03), collagen-I (R: 0.77, *p* = 0.01) and collagen-VI (R: 0.62, *p* = 0.01) and the number of M2 macrophages (R: 0.67, *p* = 0.03) and, it had a negative correlation with the number of SCs (R: − 0.81, *p* < 0.01) and the number of capillaries (R: − 0.73, *p* = 0.02). As these growth factors were observed either inside fibers or in the stroma, we were interested in studying their expression only inside the fibers. Interestingly, we observed that the percentage of fibers expressing CTGF was increased in patients with a higher amount of fat (Fig. 4a,b). Fibers expressing CTGF, PDGFAA or TGFβ were either MYH3 + or MYH3-, and we did not see any clear correlation between the expression of these two factors in the same muscle fiber.

To see if there were differences in the expression of growth factors and ECM components in the stroma of patients and controls, we decided to analyze just the stroma without the muscle fibers. As expected, we found the content of collagen III within the stroma was higher in controls and decreased as the disease progressed (R: − 0.80, *p* = 0.01) (Fig. 4e). In contrast, collagen I and collagen-VI increased in dystrophic samples (Fig. 4e,f). We observed an increase in the CTGF, PDGFAA, and TGFβ levels in the stroma in dystrophic samples, but that was only significant for CTGF.

### Comparison between fibrotic and non-fibrotic areas in the muscle

**Figure 5:**
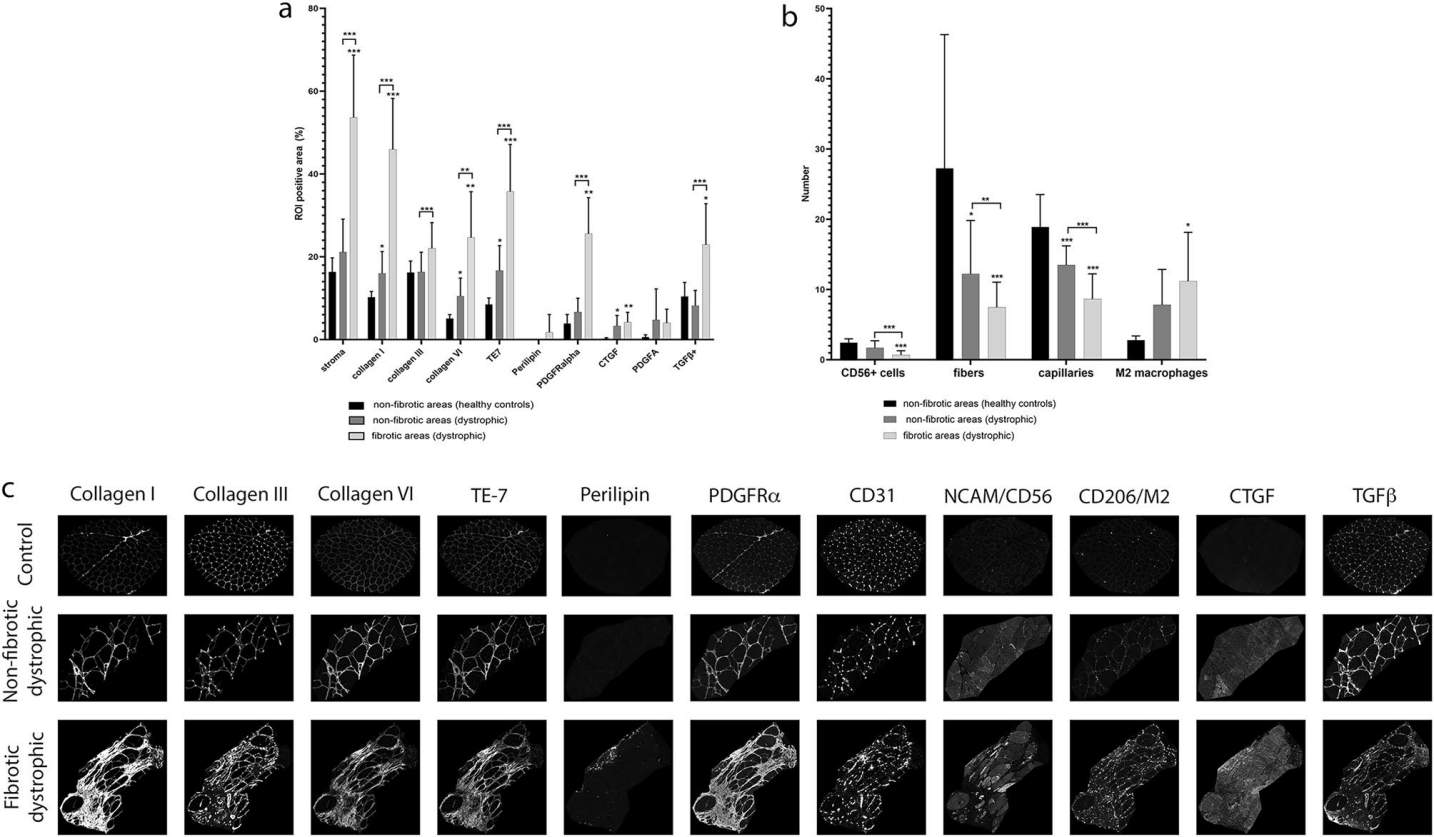
Comparison between fibrotic and non-fibrotic areas of the same or different samples. (**a**) Mean of the positive area (%) occupied by stroma, collagen I, collagen III, collagen VI, TE7, perilipin, PDGFRalpha, CTGF, PDGFA and TGFβ in non-fibrotic areas from healthy controls (n = 4, *black bars*), non-fibrotic areas from dystrophic muscles (n = 21, *dark grey bars*) and fibrotic areas from fibrotic muscles (n = 25, *light grey bars*). (**b**) Mean of number of CD56 + cells, fibers, capillaries and M2 macrophages in non-fibrotic areas from healthy controls (*black bars*), non-fibrotic areas from dystrophic muscles (*dark grey bars*) and fibrotic areas from fibrotic muscles (*light grey bars*). (**c**) IMC images representing fibrotic and non-fibrotic areas showing capillaries, ECM proteins and cells of study. Arrows point to areas where collagen I, collagen VI, TE7, perilipin, PDGFR-alpha, NCAM, CD206, CTGF and TGF-beta is increased. Error bars represent ± SD. *p ≤ 0.05, **p ≤ 0.01, ***p ≤ 0.001.

We decided to draw subregions of interest to assess the fibrotic foci and compare them with non-fibrotic areas in control and dystrophic samples. Figure 5c show a representative image of these comparisons. We did not observe significant differences in the percentage of total stroma observed between non-fibrotic areas in controls and dystrophic patients. As expected there was a clear increase in the stroma in the fibrotic regions, (*p* < 0.001) (Fig. 5a) with increased content of collagen-I (*p* < 0.001) and VI (*p* < 0.001) compared to non-fibrotic areas. We also observed a mild increase of collagen-I (*p* = 0.04), collagen VI (*p* = 0.025) and TE7 (*p* = 0.014) in the non-fibrotic foci of dystrophic patients compared to controls (Fig. 5a). The number of SCs was decreased in fibrotic areas of patients compared to controls (*p* < 0.001) and to non-fibrotic areas of patients (*p* < 0.001). However, there were not differences in SCs number between controls and non-fibrotic areas of patients. Moreover, we observed a decrease in the number of fibers and capillaries in fibrotic and non-fibrotic areas from patients compared to controls (*p* = 0.01 and *p* = 0.003, respectively). There was also an increase in the number of M2 macrophages and PDGFRα area both in the non-fibrotic and fibrotic area of the dystrophic muscles (*p* = 0.02) (Fig. 5b). Finally, we observed an increase in the expression of CTGF (*p* = 0.026) already in the non-fibrotic areas in dystrophic patients compared to controls, but not of PDGF-AA and TGFβ.

## Discussion

Muscle degeneration is a highly complex process that involves a series of actors interacting locally in what is known as the degenerative niche. These actors include muscle fibers, SCs, FAPs, macrophages, endothelial cells, and the extracellular matrix, among others. The way how these different actors interplay in humans is not well known. Here we have described the changes observed in skeletal muscle architecture and composition in samples of patients with BMD throughout disease progression using the Hyperion™ Imaging System. We have identified that infiltration of muscle by M2 macrophages, expansion of FAP cells population and release of profibrotic cytokines, especially CTGF, to the stroma are already present in apparently normal areas or with only mild increase in ECM. The progression of the disease is characterized by an expansion of the ECM, a reduction of the area occupied by muscle fibers, an increase in the proportion of regenerative fibers, loss of capillaries, and decreased number of SCs. ECM composition changes from control to disease status, being collagen-I the main component in patients while collagen-III is predominant in controls. Moreover, expansion of fat tissue is another consequence of the process of muscle degeneration, although to a less extent that increase in the ECM until very advanced stages of the disease.

BMD is produced by mutations in the dystrophin gene leading to a reduced expression of dystrophin, an essential structural protein of the skeletal muscle fiber mainly involved in the transmission of forces from inside the muscle fiber toward the ECM^1^. Muscle fibers with a reduced expression of dystrophin are susceptible to muscle contraction induced damage that triggers a series of cellular responses that may be protective or pathological^2^. On the one hand, the sarcolemma repair system mediated by the dysferlin complex is activated leading to membrane resealing, but on the other hand, calcium accumulates inside the muscle fibers either entering through the membrane tears or through the opening of calcium-permeable channels, such as transient receptor potential cation channel subfamily V member 2 (TRPV2), activating intracellular proteases such as calpains that degrade muscle membrane proteins^2^. Free radicals also play a role in the muscle fiber degradation process. After muscle damage SCs activate and proliferate aiming to regenerate muscle fibers^2,3^. In fact, we have observed an increase in the number of muscle fibers positive for MYH3, which identifies fetal myosin heavy chain expressed by regenerative fibers, but also CD56/NCAM positive fibers, which probably are immature fibers in the process of being reinnervated^25^. Interestingly, it has been suggested that injured fibers release cytokines that can also contribute to the proliferation of other muscle resident cell types, such as SCs or FAPs, and attract inflammatory cells from the bloodstream. We have observed an increase in profibrotic cytokines, especially CTGF, either in the stroma and/or inside both regenerative and non-regenerative muscle fibers, as has already been reported^26^. These results, however, need to be considered carefully, as staining is not the best way to study the expression of a soluble factor in the tissue and could be the reason why we do not see a strong TGFβ signal, which has been classically postulated as the main molecule driving fibrosis in muscular dystrophies. Nevertheless, CTGF has also been involved in the process of muscle degeneration, enhancing the proliferation of FAPS, the expansion of the ECM, and influencing the switching of M1 into M2 macrophages in the muscle, contributing to perpetuate the fibrotic environment^27^. These findings led to the development of pamrevlumab, an anti-CTGF human monoclonal antibody that is being tested in a phase-II clinical trial in patients with Duchenne Muscular Dystrophy (DMD) (https://www.clinicaltrials.gov/ct2/show/NCT02606136).

Interestingly, we have observed a progressive decrease in the number of SCs throughout disease progression that correlates with the increase in the stroma. These data might support the hypothesis that in muscular dystrophies there is an exhaustion of the regenerative capacity of the dystrophic muscle after many cycles of degeneration and regeneration, which could be due to a defective capacity of SCs to proliferate and differentiate in a fibrotic environment, or a depletion in the SCs pool due to an aging effect involving shortening of the cell telomeres produced by multiple cell divisions^28,29^.

Another interesting change observed through disease progression is the reduction in the number of vessels, especially capillaries, as observed in the moderate and advanced stages of the disease in our samples. It is not clear why there is a progressive loss of vessels in patients, however the loss of vessels could contribute to the pathogenesis of the disease. For example, it is well known that SCs are commonly found close to capillaries in the skeletal muscles, and that Vascular endothelial growth factor (VEGF), mainly released by endothelial cells stimulates myoblast migration and survival, protects myogenic cells from apoptosis, and promotes myogenic cell growth in vitro^30^. Therefore, the loss of capillaries observed here might have a negative impact in the population of SCs. Interestingly it has been suggested that SCs release VEGF to promote endothelial cell renewal although aged or dystrophic SCs have a reduced capacity to promote angiogenesis in vitro through a reduced release of angiogenic factors^31,32^. It is not well understood if a reduced number of capillaries plays a role in the process of muscle degeneration, but it is tempting to hypothesize that it can be associated with local ischaemia which is known to mediate muscle atrophy in other neuromuscular diseases such as dermatomyositis^33^. In this sense, hypoxia is being postulated as mediator of muscle damage and fibrosis in muscle dystrophy samples, a mechanism that could involve hypoxia-inducible factor-1α (HIF-1α)^34^. In this sense, a recent study has identified increased expression of CTGF by muscle fibers mediated by HIFα, suggesting that a reduced number of vessels might enhance muscle fibrosis in patients with muscular dystrophies^35^. Moreover, the vessel wall is also the home of muscle resident mesenchymal stem cells, such as pericytes and Pw1 interstitial cells, which have also been involved in the process of muscle regeneration in DMD animal models and patients suggesting that a loss of capillaries could be associated with a reduced population of MSC capable to contribute to muscle regeneration^36–38^.

An increase in the population of FAP cells is probably the main cause of the expansion of the ECM. Interestingly, we have observed a change in the composition of the matrix in BMD patients characterized by an increase in collagen I and VI, as has already been reported^39^. It has been suggested that collagen-I can significantly inhibit myogenic differentiation, although this is not completely clear as there are studies suggesting that collagen-I could contribute to the proliferation and migration of myoblasts^40–42^. There is only scarce information about the effect on muscle resident cells of an increase of collagen-VI although it has been shown that apart of FAPs other cell types can contribute to its deposition, such as M2 macrophages^37,38^. In any case, the changes observed in the ECM composition could modify matrix biophysical properties, through for example an increase in stiffness which has been suggested to influence cell functions like proliferation, migration, and differentiation^43–46^.

We have observed that accumulation of ECM occurs in foci spread all over the tissue. The detailed study of fibrotic and non-fibrotic foci in patients revealed interesting findings. First, there is an increase in M2 macrophages already happening since early stages of fibrosis associated with an increase in cytokines expression within muscle fibers, especially CTGF. Second, PDGFR-α and TE7 staining increased suggesting that FAPs and fibroblasts population expands and may contribute to the increase of the ECM. Third, even though we selected areas with no clear increase in the stroma, we already observed an increase in collagen-I and collagen-VI, suggesting that apart from the existence of fibrotic foci in the biopsy, there is already a widespread expression of collagen across all muscle samples. On the contrary, fat deposition seems to be focal in early to moderate stages of the disease starting at perivascular areas close to big vessels. It has been suggested that FAPs, which have also been described to be in close relation to the vessels, differentiate into adipocytes in the presence of specific stimuli influencing Notch, Wnt and Hedgehog molecular pathways, among others. However, whether all FAPs can differentiate into fat in vivo, or whether this feature is specific to a subpopulation of these cells remains to be known. The accumulation of fat in our samples was relatively low compared with the increase in fibrotic tissue which is one of the limitations of the study. However, we have included in the study two samples that were characterized by a massive replacement of the muscle tissue by fat. We were not able to identify samples with an intermediate stage which could have clarified what are the factors leading to adipose expansion or if there are differences in the cell populations present in these samples.

Hyperion technology enabled the study of many markers in the same slice of a muscle biopsy allowing to establish spatial correlations and suggest potential temporal changes in the expression of the different markers studied. This technology can contribute to study disease mechanisms in patients affected by rare diseases, where tissue samples are precious and scarce. However, the technology has a lot of limitations. There is only a minority of commercial antibodies that can be used, as they should not contain albumin. As the staining needs to happen with all the antibodies at the same time, the fixation process of the samples can only be one, limiting the quality of the signal observed with some markers. The costs are still very high, reducing the number of slides that can be studied which influenced the number of samples that we could assess, which is one the limitations of our study. Finally, as there is not a commercial software for the analysis we had to develop our own tool called HIPO to analyze the samples.

In summary, in the present study we have investigated the process of muscle degeneration taking place in BMD patients at different stages of disease progression. Our results allow us to propose a longitudinal sequence of events starting from the release of profibrotic cytokines to the stroma, followed by an increase in FAPs and M2 macrophages leading to accumulation of collagen-I and VI in foci leading a progressive reduction in the number of vessels and SCs. Fat tissue, that started in foci surrounding vessels, expanded massively in advanced cases when most of the muscle fibers were lost. The results presented, if confirmed in larger studies, could help to confirm the sequence of events taking place in the process of muscle degeneration and contribute to the monitor of therapies aiming to treat muscular dystrophies.

### Supplementary Information

Supplementary Figures.

## Data Availability

The raw data of the analysis obtained as well as the images acquired are available for further research upon reasonable request to the corresponding author. Hypo, the software designed for the analysis of IMC images, is available for use for free through collaboration agreements between researchers and the corresponding author.

## References

[1] Hoffman EP, Kunkel LM (1989). Dystrophin abnormalities in Duchenne/Becker muscular dystrophy. Neuron..

[2] Wallace GQ, McNally EM (2009). Mechanisms of muscle degeneration, regeneration, and repair in the muscular dystrophies. Annu. Rev. Physiol..

[3] Bencze M (2022). Mechanisms of myofibre death in muscular dystrophies: The emergence of the regulated forms of necrosis in myology. Int. J. Mol. Sci..

[4] Cappellari O, Mantuano P, De Luca A (2020). “The social network” and muscular dystrophies: The lesson learnt about the niche environment as a target for therapeutic strategies. Cells.

[5] Chang NC, Rudnicki MA (2014). Satellite cells: The architects of skeletal muscle. Curr. Top. Dev. Biol..

[6] Dumont NA, Wang YX, von Maltzahn J, Pasut A, Bentzinger CF, Brun CE (2015). Dystrophin expression in muscle stem cells regulates their polarity and asymmetric division. Nat. Med..

[7] Boldrin L, Zammit PS, Morgan JE (2015). Satellite cells from dystrophic muscle retain regenerative capacity. Stem Cell Res..

[8] Joe AW, Yi L, Natarajan A, Le Grand F, So L, Wang J (2010). Muscle injury activates resident fibro/adipogenic progenitors that facilitate myogenesis. Nat. Cell Biol..

[9] Pinol-Jurado P, Gallardo E, de Luna N, Suarez-Calvet X, Sanchez-Riera C, Fernandez-Simon E (2017). Platelet-derived growth factor BB influences muscle regeneration in duchenne muscle dystrophy. Am. J. Pathol..

[10] Serrano AL, Munoz-Canoves P (2010). Regulation and dysregulation of fibrosis in skeletal muscle. Exp. Cell Res..

[11] Lemos DR, Babaeijandaghi F, Low M, Chang CK, Lee ST, Fiore D (2015). Nilotinib reduces muscle fibrosis in chronic muscle injury by promoting TNF-mediated apoptosis of fibro/adipogenic progenitors. Nat. Med..

[12] Chen W, You W, Valencak TG, Shan T (2022). Bidirectional roles of skeletal muscle fibro-adipogenic progenitors in homeostasis and disease. Ageing Res. Rev..

[13] Biferali B, Proietti D, Mozzetta C, Madaro L (2019). Fibro-adipogenic progenitors cross-talk in skeletal muscle: The social network. Front. Physiol..

[14] Hatton CF, Botting RA, Duenas ME, Haq IJ, Verdon B, Thompson BJ (2021). Delayed induction of type I and III interferons mediates nasal epithelial cell permissiveness to SARS-CoV-2. Nat. Commun..

[15] Petrany MJ, Swoboda CO, Sun C, Chetal K, Chen X, Weirauch MT (2020). Single-nucleus RNA-seq identifies transcriptional heterogeneity in multinucleated skeletal myofibers. Nat. Commun..

[16] Dubuisson N, Versele R, Planchon C, Selvais CM, Noel L, Abou-Samra M (2022). Histological methods to assess skeletal muscle degeneration and regeneration in duchenne muscular dystrophy. Int. J. Mol. Sci..

[17] Giesen C, Wang HA, Schapiro D, Zivanovic N, Jacobs A, Hattendorf B (2014). Highly multiplexed imaging of tumor tissues with subcellular resolution by mass cytometry. Nat. Methods.

[18] FCS express software version 7, Dotmatics, USA. https://denovosoftware.com

[19] MCD viewer software, v1.0.560.6, 12 Aug 2021. https://www.standardbio.com/products/software

[20] Hunter, B., Nicorescu, I., Foster, E., McDonald, D., Hulme, G., Thomson, A., Hilkens, C.M.U. *et al.* OPTIMAL: And optimised imaging mass cytometry analysis framework for segmentation and data exploration. *bioRxiv* (2023). 10.1101/2023.02.21.526083 10.1002/cyto.a.24803 PMC10952805 37750225

[21] HIPO Software version 1.0, 2022 https://github.com/Jose-Verdu-Diaz/hipo.

[22] Qu-Path software version 0.4.3. https://qupath.github.io, version 0.4.3

[23] Napari Software version 0.4.18. https://napari.org/stable/

[24] Bayram B, Thaler R, Bettencourt JW, Limberg AK, Sheehan KP, Owen AR (2022). Human outgrowth knee fibroblasts from patients undergoing total knee arthroplasty exhibit a unique gene expression profile and undergo myofibroblastogenesis upon TGFbeta1 stimulation. J. Cell Biochem..

[25] Covault J, Sanes JR (1985). Neural cell adhesion molecule (N-CAM) accumulates in denervated and paralyzed skeletal muscles. Proc. Natl. Acad. Sci. U. S. A..

[26] Morales MG, Acuna MJ, Cabrera D, Goldschmeding R, Brandan E (2018). The pro-fibrotic connective tissue growth factor (CTGF/CCN2) correlates with the number of necrotic-regenerative foci in dystrophic muscle. J. Cell Commun. Signal..

[27] Rebolledo DL, Lipson KE, Brandan E (2021). Driving fibrosis in neuromuscular diseases: Role and regulation of Connective tissue growth factor (CCN2/CTGF). Matrix Biol. Plus..

[28] Heslop L, Morgan JE, Partridge TA (2000). Evidence for a myogenic stem cell that is exhausted in dystrophic muscle. J. Cell Sci..

[29] Vita GL, Aguennouz M, Sframeli M, Sanarica F, Mantuano P, Oteri R (2020). Effect of exercise on telomere length and telomere proteins expression in mdx mice. Mol. Cell Biochem..

[30] Arsic N, Zacchigna S, Zentilin L, Ramirez-Correa G, Pattarini L, Salvi A (2004). Vascular endothelial growth factor stimulates skeletal muscle regeneration in vivo. Mol. Ther..

[31] Christov C, Chretien F, Abou-Khalil R, Bassez G, Vallet G, Authier FJ (2007). Muscle satellite cells and endothelial cells: Close neighbors and privileged partners. Mol. Biol. Cell..

[32] Rhoads RP, Flann KL, Cardinal TR, Rathbone CR, Liu X, Allen RE (2013). Satellite cells isolated from aged or dystrophic muscle exhibit a reduced capacity to promote angiogenesis in vitro. Biochem. Biophys. Res. Commun..

[33] De Luna N, Suarez-Calvet X, Lleixa C, Diaz-Manera J, Olive M, Illa I (2017). Hypoxia triggers IFN-I production in muscle: Implications in dermatomyositis. Sci. Rep..

[34] Valle-Tenney R, Rebolledo D, Acuna MJ, Brandan E (2020). HIF-hypoxia signaling in skeletal muscle physiology and fibrosis. J. Cell Commun. Signal..

[35] Valle-Tenney R, Rebolledo DL, Lipson KE, Brandan E (2020). Role of hypoxia in skeletal muscle fibrosis: Synergism between hypoxia and TGF-beta signaling upregulates CCN2/CTGF expression specifically in muscle fibers. Matrix Biol..

[36] Dellavalle A, Maroli G, Covarello D, Azzoni E, Innocenzi A, Perani L (2011). Pericytes resident in postnatal skeletal muscle differentiate into muscle fibres and generate satellite cells. Nat. Commun..

[37] Diaz-Manera J, Gallardo E, de Luna N, Navas M, Soria L, Garibaldi M (2012). The increase of pericyte population in human neuromuscular disorders supports their role in muscle regeneration in vivo. J. Pathol..

[38] Pannerec A, Formicola L, Besson V, Marazzi G, Sassoon DA (2013). Defining skeletal muscle resident progenitors and their cell fate potentials. Development.

[39] Capitanio D, Moriggi M, Torretta E, Barbacini P, De Palma S, Vigano A (2020). Comparative proteomic analyses of Duchenne muscular dystrophy and Becker muscular dystrophy muscles: Changes contributing to preserve muscle function in Becker muscular dystrophy patients. J. Cachexia Sarcopenia Muscle..

[40] Goetsch KP, Kallmeyer K, Niesler CU (2011). Decorin modulates collagen I-stimulated, but not fibronectin-stimulated, migration of C2C12 myoblasts. Matrix Biol..

[41] Liu X, Gao Y, Long X, Hayashi T, Mizuno K, Hattori S (2020). Type I collagen promotes the migration and myogenic differentiation of C2C12 myoblasts via the release of interleukin-6 mediated by FAK/NF-kappaB p65 activation. Food Funct..

[42] Macfelda K, Kapeller B, Wilbacher I, Losert UM (2007). Behavior of cardiomyocytes and skeletal muscle cells on different extracellular matrix components–relevance for cardiac tissue engineering. Artif. Organs..

[43] Schnoor M, Cullen P, Lorkowski J, Stolle K, Robenek H, Troyer D (2008). Production of type VI collagen by human macrophages: A new dimension in macrophage functional heterogeneity. J. Immunol..

[44] Spencer M, Yao-Borengasser A, Unal R, Rasouli N, Gurley CM, Zhu B (2010). Adipose tissue macrophages in insulin-resistant subjects are associated with collagen VI and fibrosis and demonstrate alternative activation. Am. J. Physiol. Endocrinol. Metab..

[45] Loomis T, Hu LY, Wohlgemuth RP, Chellakudam RR, Muralidharan PD, Smith LR (2022). Matrix stiffness and architecture drive fibro-adipogenic progenitors’ activation into myofibroblasts. Sci. Rep..

[46] Stearns-Reider KM, D’Amore A, Beezhold K, Rothrauff B, Cavalli L, Wagner WR (2017). Aging of the skeletal muscle extracellular matrix drives a stem cell fibrogenic conversion. Aging Cell..

