# Supplementary material for: Imaging mass cytometry analysis of Becker muscular dystrophy muscle samples reveals different stages of muscle degeneration
Source: Sci Rep. 2024 Feb 9;14:3365. doi: 10.1038/s41598-024-51906-x (PMC10858026; doi:10.1038/s41598-024-51906-x)
Supplement: Supplementary file 1 — Supplementary Figures. [file 41598_2024_51906_MOESM1_ESM.docx]

**Title:** Imaging Mass Cytometry analysis of Becker muscular dystrophy muscle samples reveals different stages of muscle degeneration

**Authors:** Patricia Piñol-Jurado^1^, José Verdú-Díaz^1^, Esther Fernández-Simón^1^, Cristina Domínguez-González^2,9^, Aurelio Hernández-Lain^3^, Conor Lawless^4^, Amy Vincent^5^, Alejandro González-Chamorro^1^, Elisa Villalobos^1^, Alexandra Monceau^1^, Zoe Laidler^1^, Priyanka Mehra^1^, James Clark^1^, Paul Rushton^6^, Andrew Bowey^6^, Jorge Alonso Pérez^7,8^, Giorgio Tasca^1^, Chiara Marini-Bettolo^1^, Michela Guglieri^1^, Volker Straub^1^, Xavier Suárez-Calvet^7^ and Jordi Díaz-Manera*^1,7,9^

**Affiliations:**

1. John Walton Muscular Dystrophy Research Centre, Newcastle University Translational and Clinical Research Institute, Newcastle upon Tyne, United Kingdom.
2. Neuromuscular disorders Unit. Neurology department. imas12 Research Institute. Hospital Universitario 12 de Octubre, Madrid, Spain.
3. Neuropathology Unit, imas12 Research Institute. Hospital Universitario 12 de Octubre, Madrid, Spain
4. Newcastle University Translational and Clinical Research Institute, United Kingdom
5. Welcome Centre for Mitochondrial Research, Faculty of Medical Sciences, Translational and Clinical Research Institute, Newcastle University, Newcastle upon Tyne, United Kingdom.
6. Department of Orthopaedic Spine Surgery, Great North Children’s Hospital, Royal Victoria Infirmary, Newcastle upon Tyne, United Kingdom.
7. Neuromuscular Diseases Unit, Department of Neurology, Hospital de la Santa Creu i Sant Pau, Institut d’Investigació Biomèdica Sant Pau (IBB SANT PAU), Barcelona, Spain.
8. Neuromuscular Disease Unit. Neurology Department. Hospital Nuestra Señora de Candelaria. Fundación Canaria Instituto de Investigacion Sanitaria de Canarias (FIISC), Tenerife, Spain.
9. Centro de Investigación Biomédica en Red en Enfermedades Raras (CIBERER), Spain.


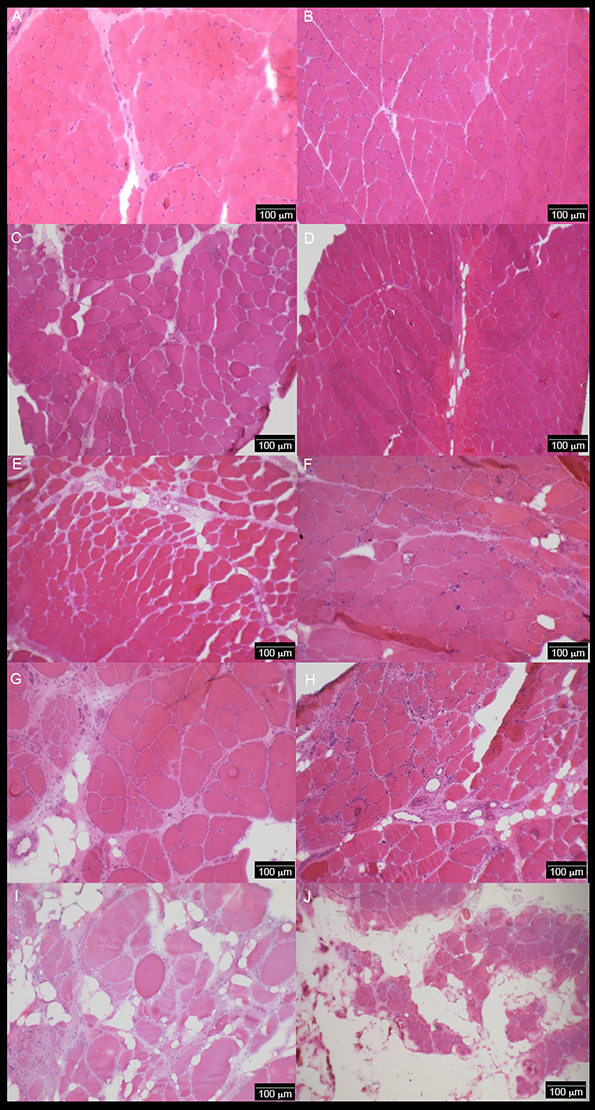


Supplemental Fig. 1: Representative microscopy (10x augmentation) images of muscle sections stained with Hematoxylin-eosin (H&E) showing different degrees of degeneration. **a** control muscle (sample C-1). **b** control muscle (sample C-2). **c** muscle with mild degeneration (BMD-1). **d** muscle with mild degeneration (sample BMD-2). **e** muscle with moderate degeneration (sample BMD-3). **f** muscle with moderate degeneration (sample BMD-4). **g** muscle with severe degeneration presenting high amount of fibrosis (sample BMD-5). **h** muscle with severe degeneration presenting high amount of fibrosis (sample BMD-6). **i** muscle with severe degeneration presenting traces of fat (represented by white holes where fat used to be before muscle processing) (sample BMD-7). **j** muscle with severe degeneration presenting traces of fat (sample BMD-8).


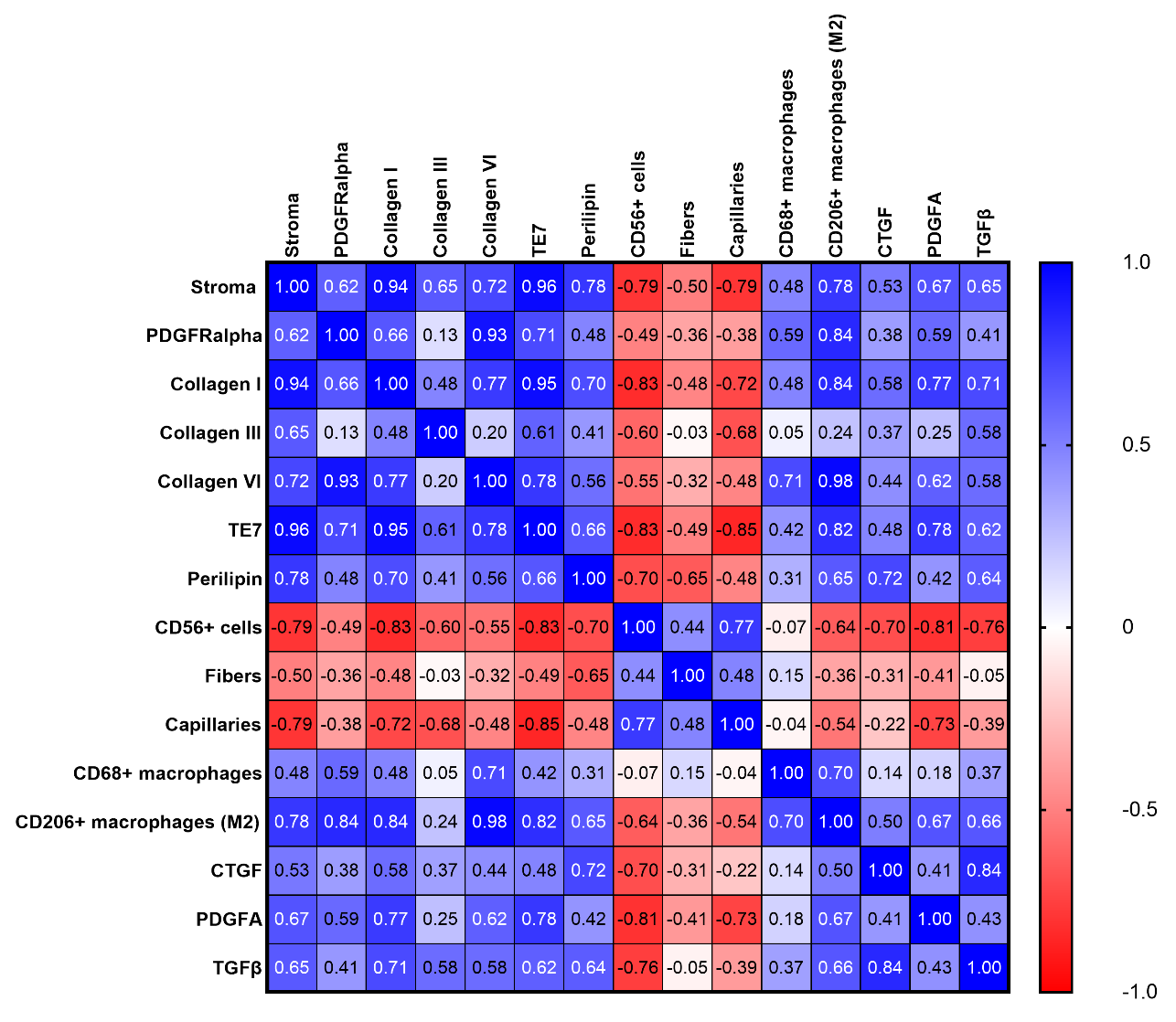


Supplemental Fig. 2: Spearman r correlation matrix displaying the correlation coefficients (r) between variables studied in the ROI without segmentation (area percentage of stroma, PDGFRalpha, collagen I, collagen III, collagen VI, TE7, perilipin CTGF, PDGFAA, TGFβ; number of cells: CD56+ cells, CD68+ macrophages and CD206+ macrophages (M2); number of fibers and number of capillaries). 1 ROI per sample.


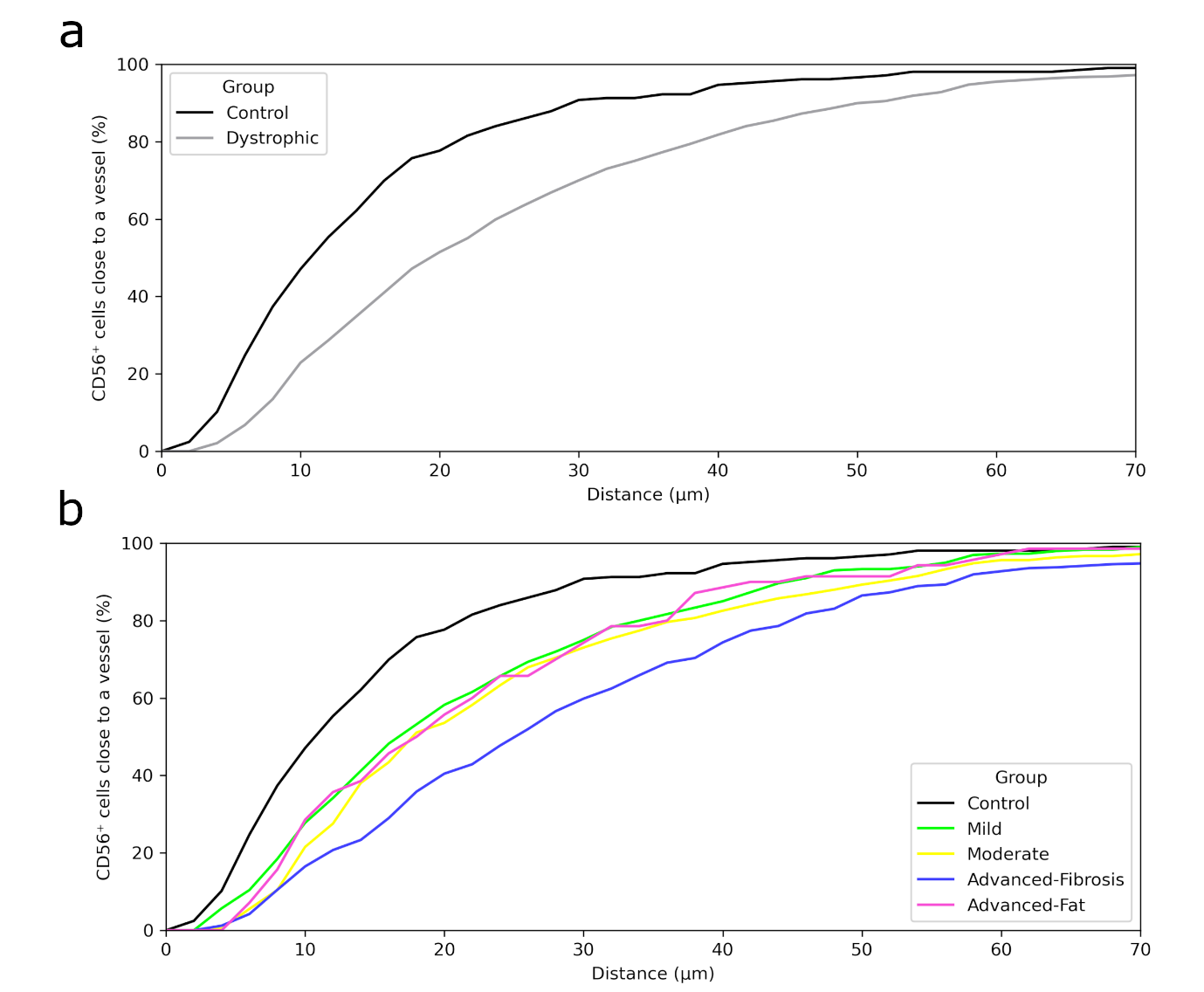


Supplemental Fig. 3: Percentage of CD56 positive cells as a function of the distance to the closest vessel, identified with CD31+ cells. **a** calculation of distances in healthy control (n=2, *black line*) and dystrophic muscles (n=8, *grey line*). **b** calculation of distances in healthy controls (n=2, *black bars*), mild (n=2, *green line*), moderate (n=2, *yellow line*), advanced-fibrosis (n=2, *blue line*) and advanced-fat conditions (n=2, *pink line*). From a distance of 15 µm the two cell types are considered not to be in contact.


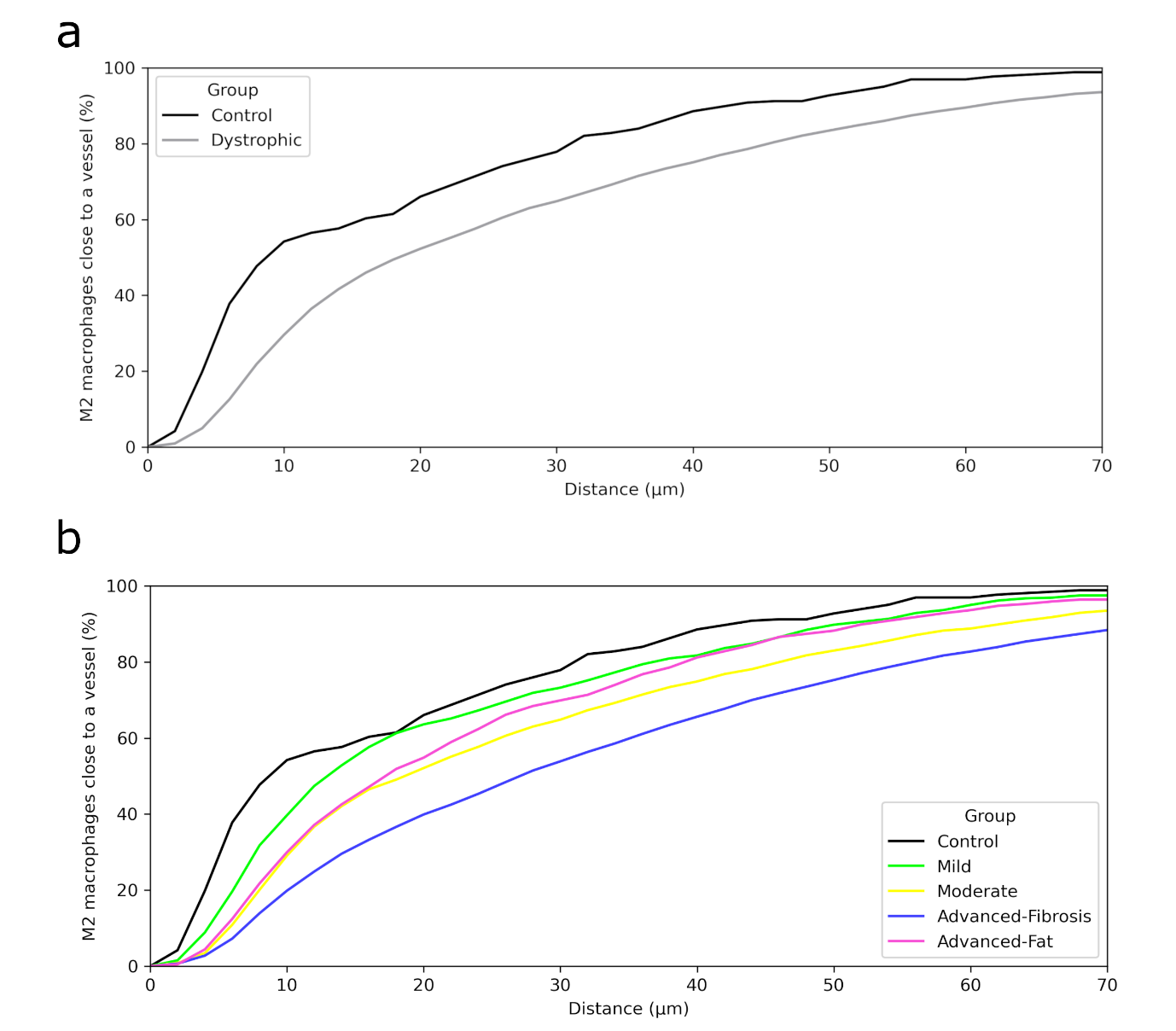


Supplemental Fig. 4: Percentage of M2 macrophages as a function of the distance to the closest vessel, identified with CD31+ cells. **a** calculation of distances in healthy control (n=2, *black line*) and dystrophic muscles (n=8, *grey line*). **b** calculation of distances in healthy controls (n=2, *black bars*), mild (n=2, *green line*), moderate (n=2, *yellow line*), advanced-fibrosis (n=2, *blue line*) and advanced-fat conditions (n=2, *pink line*). From a distance of 15 µm the two cell types are considered not to be in contact.


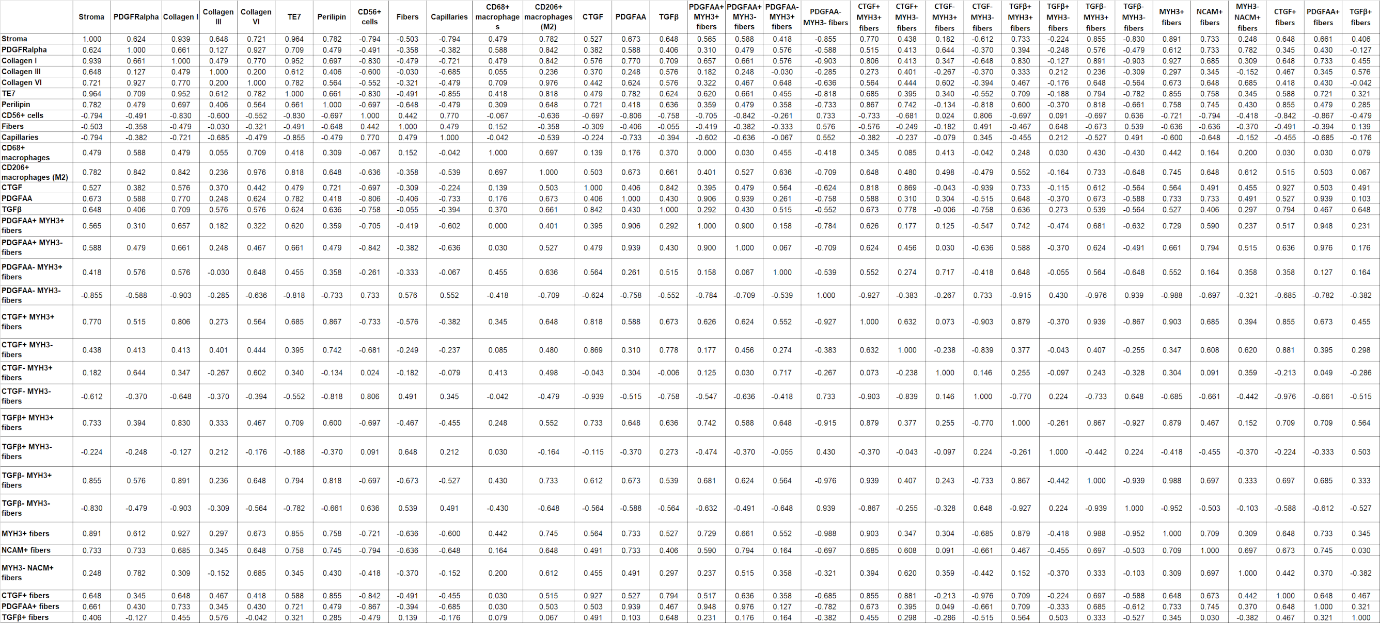


Supplemental Fig.5: Spearman r correlation matrix displaying the correlation coefficients (r) between variables studied in the entire ROI commented in supplemental Fig. 2 and the percentage of fibers positive for different proteins, after segmentation and applying a threshold for each variable.


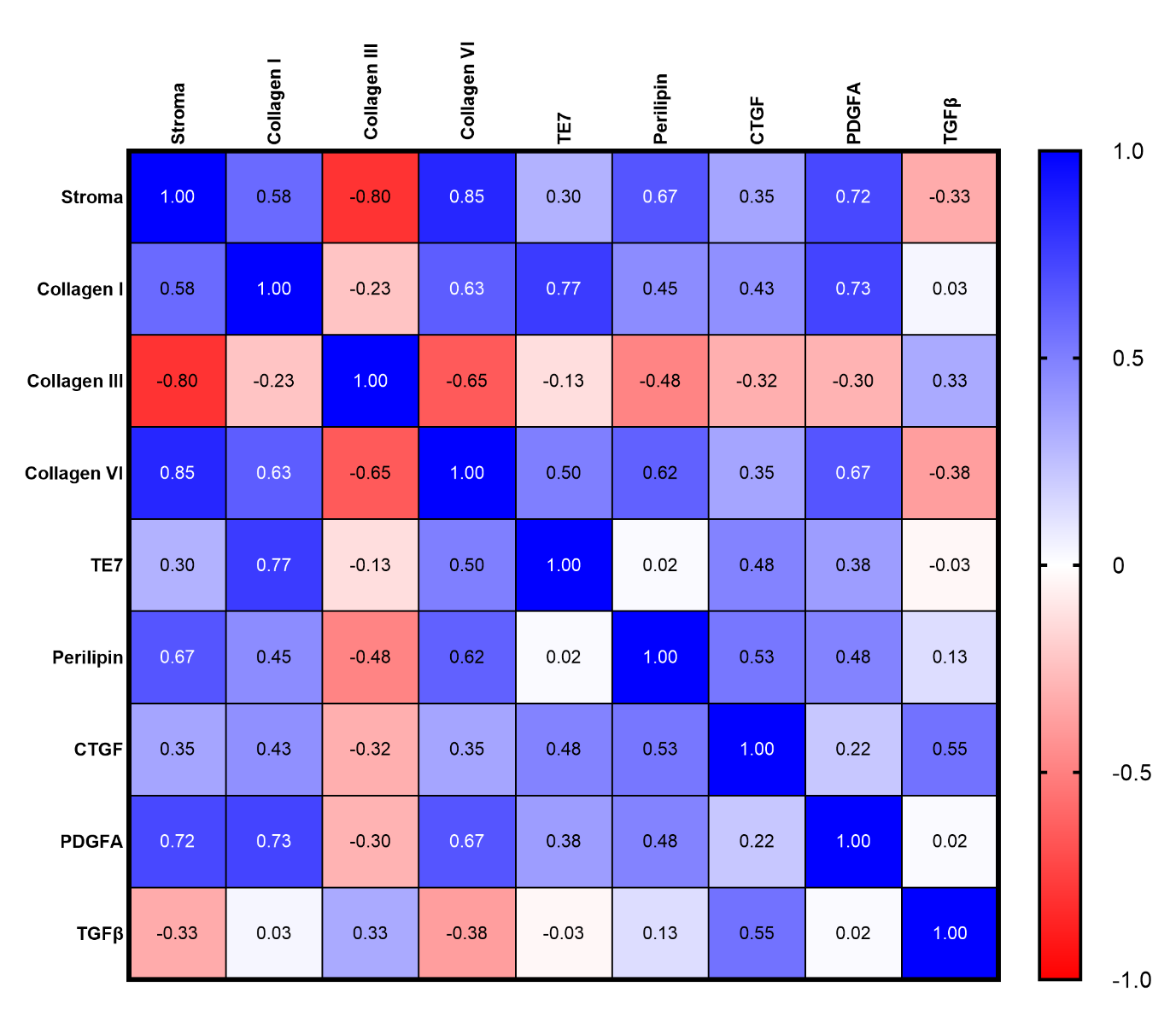


Supplemental Fig.6: Spearman r correlation matrix displaying the correlation coefficients (r) between proteins found in the stroma of the selected ROI after segmentation (collagen I, collagen III, collagen VI, TE7, Perilipin, CTGF, PDGFA and TGFβ).


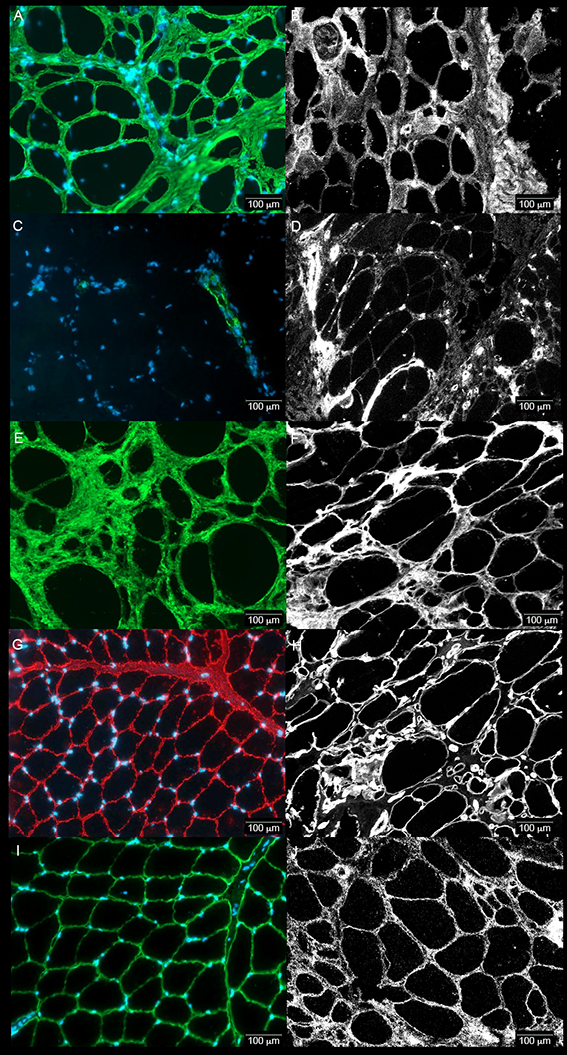


Supplemental figure 7: Validation of antibodies used for the Hyperion (I). Results obtained with immunofluorescence are shown on the left column while images obtained with Hyperion are shown on the right column. Representative images of TE7 (A-B), alpha-SMA (C-D), Collagen-I (E-F), Collagen-III (G-H) and Collagen VI (I-J).


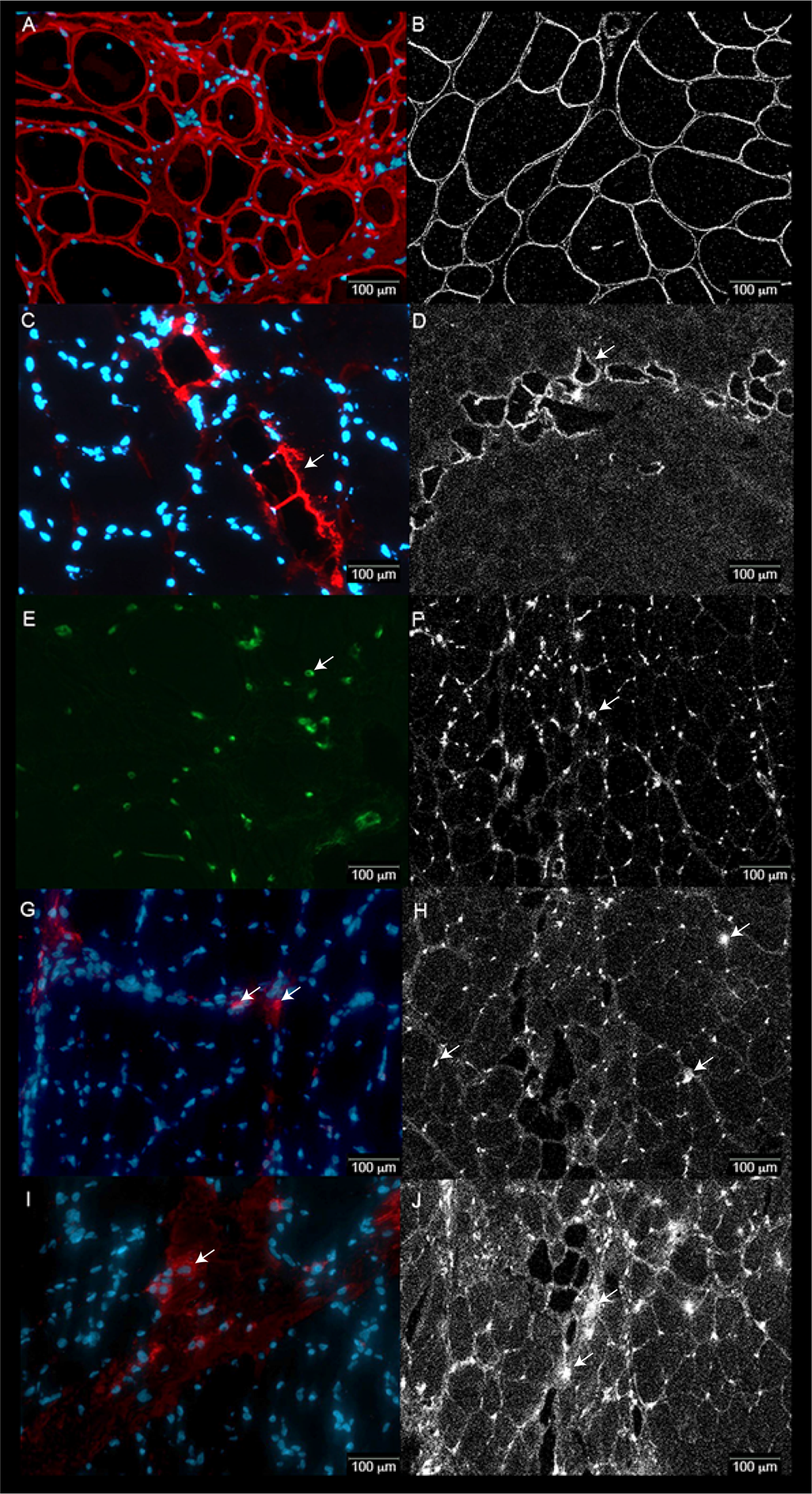


Supplemental figure 8: Validation of antibodies used for the Hyperion (II). Results obtained with immunofluorescence are shown on the left column while images obtained with Hyperion are shown on the right column. Representative images of laminin (A-B), perilipin (C-D, arrow point signal from fat vacuole wall), CD31/PECAM (E-F, arrow pointing some capillaries), CD206 (G-H, arrow pointing positive cells) and CD68 (I-J, arrow pointing some positive cells).


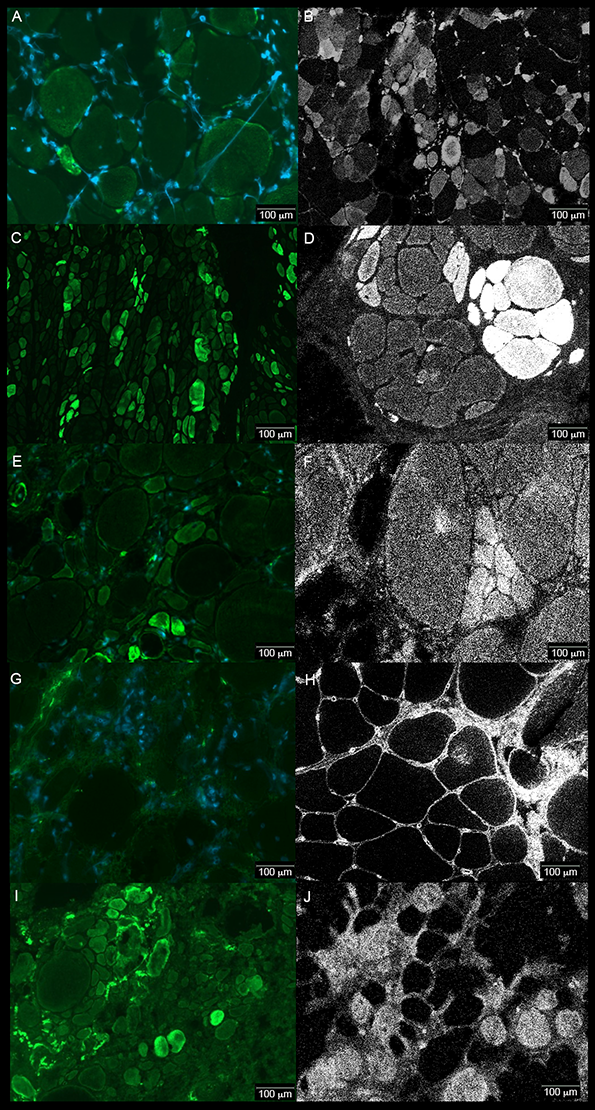


Supplemental figure 9: Validation of antibodies used for the Hyperion (III). Results obtained with immunofluorescence are shown on the left column while images obtained with Hyperion are shown on the right column. Representative images of CD56/NCAM (A-B), MYH3 (C-D,), CTGF (E-F), TGFbeta (G-H, arrow pointing positive cells) and PDGFA (I-J, arrow pointing some positive cells).


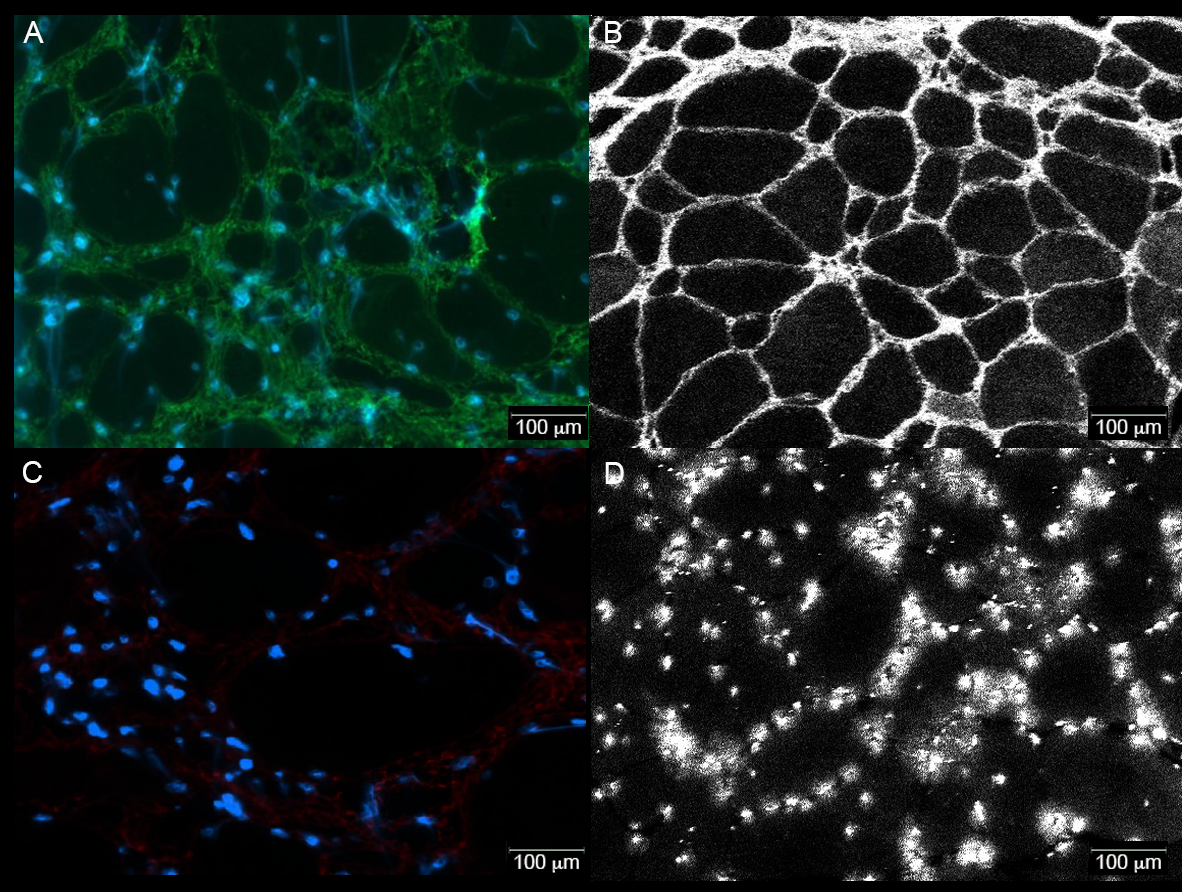


Supplemental figure 10: Validation of antibodies used for the Hyperion (III). Results obtained with immunofluorescence are shown on the left column while images obtained with Hyperion are shown on the right column. Representative images of PDGFR-alpha (A-B) and DAPI/DNA (C-D).
